# A(H2N2) and A(H3N2) influenza pandemics elicited durable cross-reactive and protective antibodies against avian N2 neuraminidases

**DOI:** 10.1038/s41467-024-49884-9

**Published:** 2024-07-03

**Authors:** Zaolan Liang, Xia Lin, Lihong Sun, Kimberly M. Edwards, Wenjun Song, Hailiang Sun, Yanmin Xie, Fangmei Lin, Shiman Ling, Tingting Liang, Biying Xiao, Jiaqi Wang, Min Li, Chin-Yu Leung, Huachen Zhu, Nisha Bhandari, Raghavan Varadarajan, Min Z. Levine, Malik Peiris, Robert Webster, Vijaykrishna Dhanasekaran, Nancy H. L. Leung, Benjamin J. Cowling, Richard J. Webby, Mariette Ducatez, Mark Zanin, Sook-San Wong

**Affiliations:** 1 HKU-Pasteur Research Pole, LKS Faculty of Medicine, The University of Hong Kong, Hong Kong SAR, China; 2 School of Public Health, LKS Faculty of Medicine, The University of Hong Kong, Hong Kong SAR, China; 3 State Key Laboratory of Respiratory Disease, Guangzhou Medical University, Guangzhou, China; 4 Guangzhou Institute for Respiratory Health and First Affiliated Hospital of Guangzhou Medical University, Guangzhou, China; 5 Guangzhou National Laboratory, No. 9 XingDaoHuanBei Road, Guangzhou International Bio Island, Guangzhou, 510005 Guangdong Province China; 6 College of Veterinary Medicine, South China Agricultural University, Guangzhou, China; 7 WHO Collaborating Centre for Infectious Disease Epidemiology and Control, School of Public Health, Li Ka Shing Faculty of Medicine, The University of Hong Kong, Hong Kong SAR, China; 8 State Key Laboratory of Emerging Infectious Diseases, The University of Hong Kong, Hong Kong SAR, China; 9 Joint Institute of Virology (Shantou University and The University of Hong Kong), Guangdong-Hongkong Joint Laboratory of Emerging Infectious Diseases, Shantou University, Shantou, P. R. China; 10 Molecular Biophysics Unit, Indian Institute of Science, Bangalore, Karnataka India; 11 US Center for Disease Control and Prevention, Atlanta, GA USA; 12 Center for Immunology & Infection, Hong Kong Science and Technology Park, Hong Kong SAR, China; 13 Department of Host-Microbe Interactions, St. Jude Children’s Research Hospital, Memphis, TN USA; 14 Laboratory of Data Discovery for Health, Hong Kong Science and Technology Park, Hong Kong SAR, China; 15 Interactions Hosts-Pathogens (IHAP), Université de Toulouse, National Research Institute for Agriculture, Food and the Environment (INRAE), National Veterinary School of Toulouse (ENVT), Toulouse, France

**Keywords:** Influenza virus, Antibodies, Viral host response, Epidemiology

## Abstract

Human cases of avian influenza virus (AIV) infections are associated with an age-specific disease burden. As the influenza virus N2 neuraminidase (NA) gene was introduced from avian sources during the 1957 pandemic, we investigate the reactivity of N2 antibodies against A(H9N2) AIVs. Serosurvey of healthy individuals reveal the highest rates of AIV N2 antibodies in individuals aged ≥65 years. Exposure to the 1968 pandemic N2, but not recent N2, protected against A(H9N2) AIV challenge in female mice. In some older adults, infection with contemporary A(H3N2) virus could recall cross-reactive AIV NA antibodies, showing discernable human- or avian-NA type reactivity. Individuals born before 1957 have higher anti-AIV N2 titers compared to those born between 1957 and 1968. The anti-AIV N2 antibodies titers correlate with antibody titers to the 1957 N2, suggesting that exposure to the A(H2N2) virus contribute to this reactivity. These findings underscore the critical role of neuraminidase immunity in zoonotic and pandemic influenza risk assessment.

## Abstract (web summary)

Using more than 300 serum samples from individuals with an age range of 1-88 years old, the authors here show that older adults possess higher breadth of antibody reactivity to avian influenza virus (AIV) neuraminidases, potentially explaining the age-specific risks of AIV human infections.

## Introduction

Introduction Avian influenza A viruses (AIVs) are of considerable concern to public health. Sporadic human outbreaks caused by 13 AIV subtypes have led to over 2739 reported cases since 1996. The recent emergence and geographical expansion of novel A(H5Nx) viruses has also raised public health concerns^1^(https://www.who.int/publications/m/item/influenza-at-the-human-animal-interface-summary-and-assessment)^2^. Notably, AIV subtypes A(H7N9), A(H5N1) and A(H9N2), responsible for the majority of human infections, exhibit distinct age-dependent incidence of infection and severe disease that have yet to be explained by current knowledge of host, viral or environmental factors. A(H5N1) AIV cases are predominant among individuals ≤15 years old (yo) while A(H7N9) AIV cases are more commonly reported in adults aged ≥60yo^2,3^. Given the increasing spread of these novel AIV subtypes, understanding factors that shape these age-specific epidemiological patterns can help to elucidate the pandemic risks associated with AIVs.

Current pandemic risk assessment frameworks used by the World Health Organization (WHO) rely on measuring neutralizing antibodies or antibodies targeting the hemagglutinin (HA) protein that are detected by the hemagglutination inhibition assay, to assess population immunity^4^. However, recent evidence suggests that antibodies targeting the neuraminidase (NA) protein exhibit broad cross-reactivity^5–8^ and play a significant role in reducing disease severity following seasonal influenza virus infections^9^. Indeed, N2 antibodies elicited by the 1957 A(H2N2) pandemic strain were postulated to have reduced the impact of the 1968 A(H3N2) pandemic^10^. However, the importance of NA antibodies in the context of AIV infections remain unexplored.

A(H9N2) AIVs are amongst the most prevalent subtype in poultry globally and have recently become the predominant circulating strain in poultry in China. This coincided with increases in cases of A(H9N2) human infections, specifically in young children^11–13^. However, seropositivity rates ranging from 1.37% to 11.2% have been observed in occupationally exposed adults in China, suggesting the occurrence of mild or asymptomatic infections in older individuals occupationally exposed to the virus^14^.

A(H9N2) AIVs share the same NA subtype as seasonal A(H3N2) influenza viruses, and the current human N2 evolved from its avian progenitor, introduced to the human population during the 1957 A(H2N2) pandemic. Because the first influenza virus exposure primes immunological memory that can be boosted upon reinfection with an antigenically-related strain later in life^15–18^, we hypothesized that cross-reactive N2 antibodies may be present in an age-dependent manner and potentially contribute to the age-specific incidence of A(H9N2) AIVs infections in humans. To investigate this, we conducted age-stratified serosurvey for antibodies against avian N2, with a focus on the A(H9N2) subtype, in cohorts of healthy individuals or individuals infected with seasonal A(H3N2). We further demonstrated the protective capacity of these cross-reactive antibodies in the mice model.

## Results

### High amino acid similarity between historical human seasonal N2 and avian N2

**Fig. 1:**
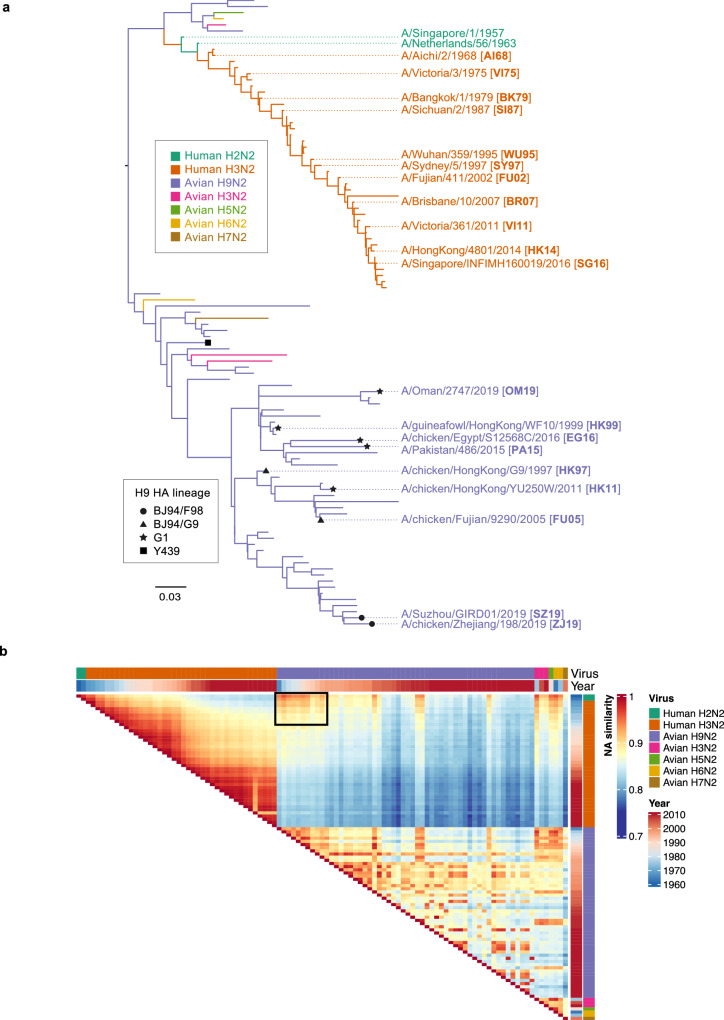
Evolutionary relationships and genetic similarity of human and avian N2 neuraminidases from 1957 to 2019. **a** Maximum likelihood phylogenetic tree with branches colored by host and subtype. Representative strains used for serological testing are labeled, and abbreviations used in this study are shown in bold. **b** Heatmap of pairwise comparison of amino acid sequence similarities for human and avian N2 strains between 1957 to 2019, generated by using “ComplexHeatmap” package in R. Annotation on the right represents the NA amino acid sequence similarities, representative strains and year of virus isolation. Highly similar sequences between avian and human N2 are boxed.

To reconstruct the evolutionary history of avian and human N2 genes, we analyzed 103 sequences representing major human A(H2N2) and A(H3N2) variants since their introduction into humans in 1957 (*n* = 42) and genetically diverse avian N2 strains collected since 1960s (*n* = 61). Figure 1a shows that human and avian N2 cluster into two distinctive branches, showing independent evolution since 1957. Human N2s evolved in a typical ladder-like pattern, suggestive of an immune driven evolution, much like HA. In contrast, avian N2s showed a more balanced phylogeny, suggesting a lack of selective pressure on the avian N2s. We found that the avian N2 from A(H9N2) AIVs consistently clustered into the same three major HA subclades: (**i**)Y439, (ii) G1, and (iii) BJ94, with BJ94 further divided into the F98 and G9 sub-lineages.

At a protein level, pairwise analysis indicated that human N2s from 1957 to early 1970s showed close to 90% amino acid sequence identity to the avian N2 strains (Fig. 1b, boxed). Human N2s gradually diverged, and viruses in the last 20 years shared only between 75% to 80% similarity with the ancestral avian N2s (Fig. 1b). These data show that until the 1970s, human N2s shared high amino acid sequence similarity with avian N2s.

### Age -specific seroprevalence and the protective capacity of the cross-reactive N2 antibodies

To assess the seroprevalence of cross-reactive avian N2 antibodies, we collected residual serum samples from healthy individuals or those admitted for reasons not related to infectious diseases to the First Affiliated Hospital of Guangzhou Medical University, located in the urban center of Guangzhou (Table 1). We used an age-stratified study design, with 20 individuals per age group, based on the expected immune imprinting profile to influenza virus strains during early life (further described in Methods). For serological testing of AIV N2 antibodies, we selected eight A(H9N2) viruses representing major A(H9N2) clades detected globally (Fig. 1a, Supplementary Table 1). We also selected 11 human A(H3N2) viruses representing major H3 antigenic types circulating from 1968 – 2016 (Fig. 1a, Supplementary Table 2)^19^.

**Table 1:** Summary of human serum samples used in this study

	Guangzhou cohort	CARES^24^	EPI-HK^25^
Location	Guangzhou, Guangdong Province	Suzhou and Yancheng, Jiangsu Province	Hong Kong
Source	First Affiliated Hospital of Guangzhou Medical University	Community	Community
Collection period	March to December 2020	2015 to 2017	2020 and on-going
Study design	Cross-sectional serosurvey of 6 different age groups: ≤5yo, 6-10, 11-20, 21-39, 40-64 and > 65 years old	Community-based study on respiratory virus infections in older adults. Serum samples were collected in 6 months interval, with active surveillance for acute respiratory illness.	Community-based longitudinal study on respiratory virus infections in individuals of all ages.
Total number of samples selected for testing	120 serum samples (*n* = 20 per age group)	86 serum samples	112 serum samples in 6 different age groups: 0-10 (*n* = 3), 11-20 (*n* = 21), 21-39 (*n* = 19), 40-64 (*n* = 20) and 65-74 (*n* = 30) and > 75 years old (*n* = 19).
Samples	Single serum sample from individuals seeking physical examination or treatment for non-infection related causes	Pre- and post- infection serum from 43 individuals with laboratory confirmed A(H3N2) infections	Baseline serum samples collected upon enrollment when EPI-HK was initiated in July 2020
Gender ratio	Prespecified at 1:1	16 males, 27 females	54 males, 58 females
Age range (years old)	1 to 82	60 to 88	6 to 83
Influenza Vaccination history	Unknown	0/43 (0%)	Unknown
Poultry exposure	Unknown	16 (37.2%) reported exposure to live poultry markets in the past 12 months	Unknown
Baseline HI titer > 40 to H7N9	Unknown	0/43 (0%)	Unknown
Baseline HI titer > 40 to H9N2	0/120	5/43	7/112

**Fig. 2:**
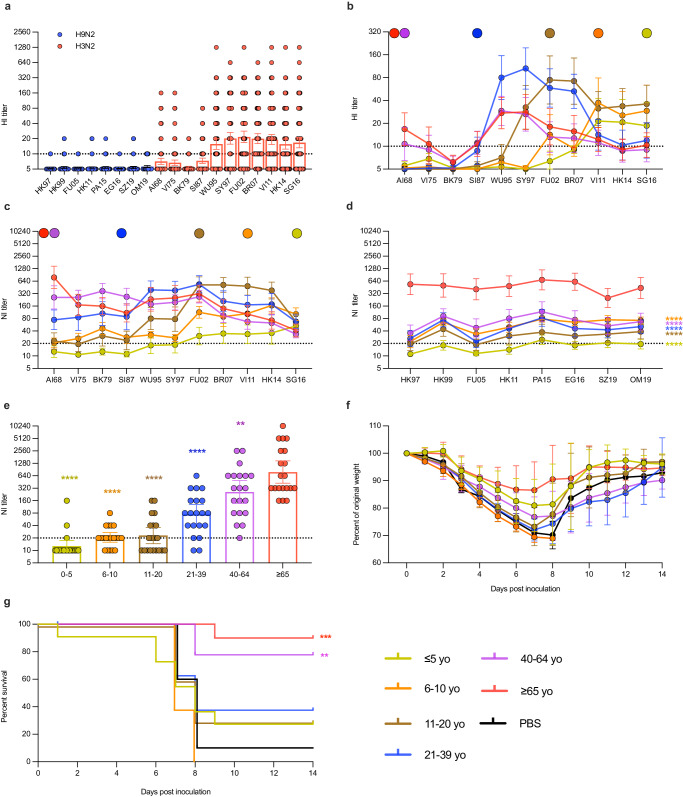
Cross-reactive and protective potential of human N2 antibodies against subtype A(H9N2) influenza A viruses in Guangzhou cohort samples. **a** Hemagglutination-inhibition (HI) titers against representative strains of subtype A(H9N2) AIV and antigenically-distinct human A(H3N2) influenza viruses in human circulation since 1968. Age-stratified **b** HI and **c** neuraminidase-inhibition (NI) antibody profiles against selected human A(H3N2) viruses. **d** Age-stratified NI-antibody profile against eight A(H9N2) AIV isolated between 1997 to 2015. **e** Age-stratified NI-titers against the 1968 pandemic strain, A/Aichi/2/1968 (H3N2) (AI68). **f** Weight loss and **g** survival curves of mice inoculated with pooled human sera from respective age-groups. All NI-antibody was detected using enzyme-linked lectin assay (ELLA) using recombinant viruses bearing the target NA with a HA gene from A/teal/Hong Kong/W312/1997 (H6N1) and the internal genes of A/Puerto Rico/8/1934 (H1N1) (PR8). Colored circles above the graph indicate those viruses circulating at the time of birth of the oldest participant in each age group. Dotted lines in (**a**–**e**) indicate limits of detection. The bar graphs indicate the geometric mean antibody titer with 95% confidence intervals, with *n* = 20 individuals per age group. Statistical significance in (**d**) was calculated using two-sided two-way ANOVA compared to ≥ 65-year-old age group using Dunnett’s multiple comparison test (****p < 0.0001 for all age groups). Statistical significance in (**d**) was calculated using two-sided two-way ANOVA compared to ≥ 65 yo group, adjusted with Dunnett’s multiple comparison test (****p < 0.0001 for all age groups). Statistical significance in (**e**) was calculated using two-sided one-way ANOVA compared ≥ 65 yo group, adjusted with Dunnett’s multiple comparison test (****p < 0.0001 for ≤ 5 yo, 6–10 yo, 11–20 yo and 21–39 yo, **p = 0.0057 for 40–64 yo). Weight loss was expressed as mea*n* ± standard deviation. Survival rate in **g** was compared using two-sided Gehan-Breslow-Wilcoxon test with PBS as the reference group (**p = 0.0024 for 40–64 yo, ***p = 0.0002 for ≥ 65 yo). Each group had *n* = 10 mice, except for the 6–10 yo group and 21–39 yo group which had 8 mice, and the 40-64 yo group which had 9.

HI titers were detectable against seven of the more recent A(H3N2) viruses but were undetectable against A(H9N2) (Fig. 2a, Supplementary Table 3) in our Guangzhou cohort. Stratifying the responses by age group revealed that peak HI and NI titers were detected against virus strains circulating within the decade of birth (Fig. 2b, c, Supplementary Tables 3 and 4), consistent with the principles of immunological imprinting. NI titers were higher than HI titers for the corresponding viruses, likely due to differences in assay sensitivity (Fig. 2b, c). However, NI titers against avian N2s were relatively consistent across the eight strains in an age-dependent manner (Fig. 2d). The oldest group, ≥65yo, had the highest titers to all eight strains, while the youngest group, ≤5yo, had undetectable or relatively low NI titers. With the exception of the 6-10yo group, NI titers against all eight N2s increased in an age-dependent manner. Compared to responses in the ≥65yo group, there was also a significant age-dependent decrease in NI titers to early human N2, represented by the pandemic virus A/Aichi/2/1968 (H3N2) (AI68) (Fig. 2e). Notably, while the ≥65yo group showed strong positive correlations between NI titers to AI68 and all eight avian N2s (r = 0.70 to 0.89, *p* < 0.0005), the 40-64yo group showed no or varying degree of correlations (r = 0.15 to 0.71, ns or *p* < 0.05-0.0005) (Supplementary Table 5). Taken together, NI titers to avian N2s were highest in older age groups and correlated with the AI68 N2 titers.

To determine whether these cross-reactive N2 antibodies were protective, we passively transferred the pooled, age-stratified sera into BALB/c mice and challenged these mice with A/chicken/Zhejiang/198/2019 (H9N2) (ZJ19). ZJ19, belonging to the BJ94/F98 clade, was pathogenic in mice without prior adaptation and is 99% similar at the nucleotide level to the human isolate of H9N2, A/Suzhou/GIRD01/2019 (SZ19) used in the serological testing. NI titers of the sera pooled from each age group used to inoculate mice were as follows: undetectable (<10) in ≤5yo, 20 in 6-10yo, 10 in 11-20yo, 20 in 21-39yo and 40-64yo, and 160 in ≥65yo. Post challenge, serum from ≥65yo individuals was the most protective, resulting in the least weight loss (Fig. 2f) and significantly better survival rates compared to PBS control mice (Fig. 2g). Although weight loss curves were similar, survival rates were also significantly higher in mice that received sera from the 40-64yo compared to PBS control. No statistically significant differences in the survival rates were observed for the other age groups. These findings suggest that individuals ≥65yo and, to some extent, those ≥40yo had cross-reactive antibodies that were protective against a lethal A(H9N2) AIV challenge.

To further exclude the role of HA-binding antibodies in our observation, whether from past H9N2 exposure or any cross-reactive antibodies that may target H9, we measured their H9-binding antibodies against a recombinant H9 protein, A/Hong Kong/1073/99 (H9N2) by ELISA. Some individuals above the ages of 21 showed H9-binding positivity (Supplementary Fig. 1a). However, except for those aged ≥ 65yo compared to the 11-20yo and the ≤ 5yo, there were no statistically significant differences amongst other age groups. Because pooled sera from the 21-39 yo did not protect the mice from the ZJ19 challenge (Fig. 2f, g), this suggests that H9-binding antibodies were not a major determinant in the protective effect observed. Further, even after excluding those participants with anti-H9 binding positivity, all our observed pattern of cross-reactivity in the Guangzhou cohort still holds (Supplementary Fig. 2), suggesting that this avian N2 cross-reactivity is not due to previous exposures to H9N2 or cross-reactive H9 antibodies.

### Antigenic relationship between early human and avian N2

**Fig. 3:**
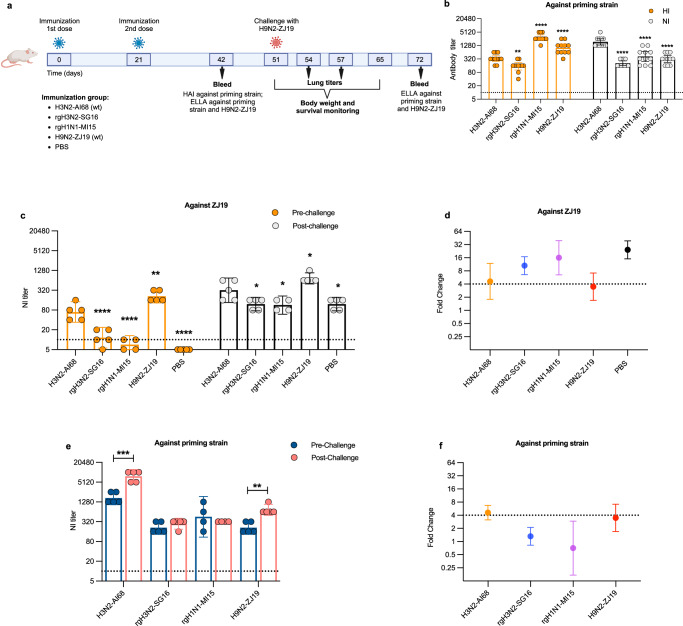
Antigenic relationship between AI68 N2 with an avian N2. **a** Experimental schema of the prime-challenge experiment. Groups of mice were immunized with two-doses of a wild-type (wt) A/Aichi/2/1968 (H3N2) (AI68), rg-derived A/Singapore/INFIMH160019/2016 (H3N2) (SG16), rg-A/Michigan/45/2015 (H1N1) (MI15) or wt-A/chicken/Zhejiang/198/2019 (H9N2) (ZJ19) and subsequently challenged with ZJ19. **b** Hemagglutination-inhibition (HI) and neuraminidase inhibition (NI) antibody profiles at Day 42 post-immunization against the priming viruses (*n* = 11 mice/group). **c** NI antibody titers of mice in each immunization group and **d** its associated fold-change against ZJ19 before and after challenge. **e** NI antibody titers of mice in each immunization group and **f** its associated fold-change against the priming viruses before and after challenge. *n* = 5 mice/group, except for MI15 which had *n* = 4. All NI-antibody was detected by enzyme-linked lectin assay (ELLA) using recombinant viruses bearing the target NA with a HA gene from A/teal/Hong Kong/W312/1997 (H6N1) and the internal genes of A/Puerto Rico/8/1934 (H1N1) (PR8). Dotted lines in **b**, **c** and **e** indicate limits of detection. The bar graphs indicate the geometric mean antibody titer with 95% confidence intervals. Statistical significance in **b**–**c** was calculated using two-sided one-way ANOVA with the H3N2-AI68 group as reference, adjusted with Dunnett’s multiple comparison test (For **b**, in HI assay, **p = 0.0026 for rgH3N2-SG16, ****p < 0.0001for rgH1N1-MI15, H9N2-ZJ19; in NI assay, ****p < 0.0001 for rgH3N2-SG16, rgH1N1-MI15, H9N2-ZJ19; for **c**, in pre-challenge **p = 0.0031 for H9N2-ZJ19, ****p < 0.0001 for rgH3N2-SG16, rgH1N1-MI15 and PBS; in post-challenge *p = 0.0112 for rgH3N2-SG16, *p = 0.0104 for rgH1N1-MI15, *p = 0.0316 for H9N2-ZJ19, *p = 0.0112 for PBS). Statistical significance between paired samples in **e** was analyzed using two-sided paired t-test (***p = 0.0004 for H3N2-AI68, **p = 0.0086 for H9N2-ZJ19). Figure 3a created with BioRender.com released under a Creative Commons Attribution-NonCommercial-NoDerivs 4.0 International license.

To determine if exposure to early human N2s was a determinant of cross-reactivity, we performed a prime-challenge experiment in mice (Fig. 3a). Mice was primed with A/Aichi/2/1968(H3N2) (AI68) wild type virus, and reverse-genetics derived viruses bearing the HA and NA of A/Singapore/INFIMH160019/2016 H3N2) (SG16) or A/Michigan/45/2015 (H1N1) (MI15). AI68 was chosen to represent the early H3N2 virus while SG16 was chosen to represent a contemporary H3N2 virus. MI15 was used as heterologous NA subtype control. Although the priming doses were predetermined to elicit comparable N2 titers between AI68 and SG16, in the eventual experiment, the two priming doses elicited higher homologous HI and NI titers in the AI68-primed mice compared to SG16 (Fig. 3b). The control ZJ19 and rgH1N1 viruses had higher HI but lower NI titers compared to AI68.

We assessed the baseline cross-reactivity in the pre-challenge immune sera to the NA of ZJ19. Apart from the positive control, AI68 had the highest titers to ZJ19 and was significantly higher compared to SG16 and MI15 (Fig. 3c). Post challenge, AI68- and ZJ19-primed groups showed smaller increases in ZJ19 N2 titers compared to SG16-, MI15- and PBS-primed groups (Fig. 3c, d), likely due to the higher levels of baseline antibody. However, only mice in the AI68 or ZJ19 group showed significant induction of priming N2 antibodies and at least four-fold increases in NI-titers after challenge (Fig. 3e, f, Supplementary Table 6). All animals were protected, with no significant differences in weight loss amongst groups (Supplementary Fig. 3a–c), except for those in the PBS group. These data indicate that AI68 and ZJ19 shared cross-reactive epitopes and were antigenically closer compared to SG16.

### AI68 immune sera were protective against ZJ19 challenge in mice

**Fig. 4:**
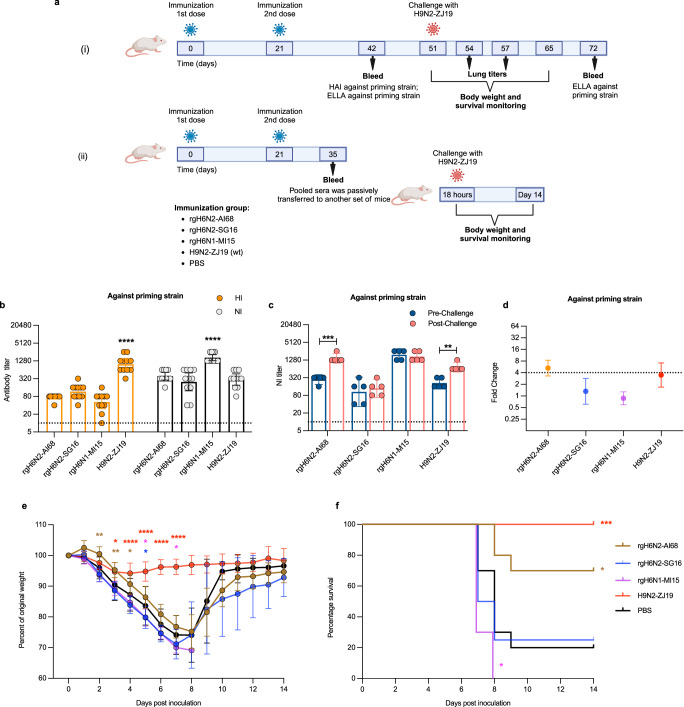
Protective capacity of the 1968 A(H3N2) pandemic N2 antibody against subtype A(H9N2) AIV infection. **a** Experimental schema of (i) prime-challenge and (ii) passive transfer experiment. Groups of mice (for **i**; *n* = 11/group, for (ii); *n* = 14/group for immunization, *n* = 10/group, except *n* = 8 for rgH6N2-SG16 group, for challenge) were immunized with two-doses of recombinant H6Nx viruses bearing the NA from A/Aichi/2/1968 (H3N2) (AI68), A/Singapore/INFIMH160019/2016 (H3N2) (SG16), or A/Michigan/45/2015 (H1N1) (MI15), or wild-type (wt) A/chicken/Zhejiang/198/2019 (H9N2) (ZJ19). From experiment (i), **b** hemagglutination-inhibition (HI) and neuraminidase inhibition (NI) antibody profiles at Day 42 post-immunization against priming strain. **c** NI antibody profiles and **d** its associated fold change against priming viruses before and after challenge with ZJ19 at Day 72. From experiment (ii), **e** weight loss and **f** survival of mice that received pooled immune sera after being challenged with ZJ19. NI-antibody was detected by enzyme-linked lectin assay (ELLA) using recombinant viruses bearing the target NA with a HA gene from A/teal/Hong Kong/W312/1997 (H6N1) and the internal genes of A/Puerto Rico/8/1934 (H1N1) (PR8). Dotted lines in (**b**) and (**c**) indicate limits of detection. The bar graphs indicate the geometric mean antibody titer with 95% confidence intervals. Statistical significance in (**b**) was calculated using two-sided one-way ANOVA using rgH6N2-AI68 group as reference, adjusted with Dunnett’s multiple comparison test (in HI assay, ****p < 0.0001 for H9N2-ZJ19; in NI assay, ****p < 0.0001 for rgH6N1-MI15). Statistical significance in (**c**) was analyzed using two-sided paired t-test (**p = 0.0086 for H9N2-ZJ19, ***p = 0.0006 for rgH6N2-AI68). Weight loss in (**e**) was expressed as mea*n* ± standard deviation, with statistical difference compared to the PBS group using two-sided, adjusted with Dunnett’s multiple comparison test from days 0 to 7 post-inoculation (days 2 *p = 0.0047 for rgH6N2-AI68; days 3 *p = 0.0106 for H9N2-ZJ19, **p = 0.0026 for rgH6N2-AI68; days 4, *p = 0.0467 for rgH6N2-AI68, ****p < 0.0001 for H9N2-ZJ19; days 5 *p = 0.0411 for rgH6N2-SG16, *p = 0.0239 rgH6N1-MI15, ****p < 0.0001 for H9N2-ZJ19; days 6, ****p < 0.0001 for H9N2-ZJ19; days 7, *p = 0.0213 for rgH6N1-MI15, ****p < 0.0001 for H9N2-ZJ19). Survival rate in (**f**) was compared to PBS group using two-sided Gehan-Breslow-Wilcoxon test (*p = 0.0128 for rgH6N2-AI68, *p = 0.0411 for rgH6N1-MI15, ***p = 0.0005 for H9N2-ZJ19). Figure 4a created with BioRender.com released under a Creative Commons Attribution-NonCommercial-NoDerivs 4.0 International license.

To clearly demonstrate the role of NA and to reduce potential interference from the different HAs, we generated a complementary set of isogenic, reverse-genetics (rg)H6Nx viruses bearing the NA from AI68, SG16, or MI15 in the second prime-challenge experiment (Fig. 4a(i)). After two priming doses, the HI titers were comparable amongst the rgH6Nx viruses but were significantly lower than the H9N2 control (Fig. 4b) whereas the NI titers were comparable for N2 but lower than the rgH6N1 control. As in Fig. 3f, challenge with ZJ19 only boosted antibodies to AI68 and its homologous antigen (Fig. 4c, d). Except for mice in the PBS group, all animals survived the challenge (Supplementary Fig. 3d–f).

To demonstrate that the antibodies elicited by the AI68 N2 were protective against ZJ19 challenge, we immunized another set of mice and conducted passive transfer of the immune sera into naïve mice prior to challenge (Fig. 4a(ii)). Mice that received SG16 and MI15 immune sera lost significantly more weight than mice receiving AI68 N2-primed sera at one to seven days post-inoculation (Fig. 4e). After day eight, surviving mice recovered their weight and were mostly indistinguishable from mice immunized with ZJ19. Despite some early weight loss, 70% of mice that received sera from AI68-primed mice survived compared to 0- 25% survival rate in the other groups (Fig. 4f). These data indicate that N2 antibodies generated by AI68 priming were protective against lethal A(H9N2) AIV infection compared to contemporary human N2.

### Cross-reactivity to other NA subtypes in older adults

We asked if these antibody responses could be recalled after seasonal influenza virus infection, and if the antibody cross-reactivity observed was limited to N2 subtypes. We used a subset of human serum samples collected from the China Ageing REspiratory infections Study (CARES), a community-based longitudinal surveillance study on respiratory viral infections in 1532 older adults, conducted between 2015-2017 in Suzhou and Yancheng, Jiangsu province, China^20^. Paired baseline and post-infection serum samples from 43 PCR-confirmed cases of A(H3N2) influenza virus infections in adults aged 60 to 88 yo were used for evaluation (Table 1). These older adults were infected with A(H3N2) viruses belonging to clade 3 c.2a. This group of selected participants has no history of influenza vaccination and 37.2% of them reported visits to live poultry market within the last 12 months. At baseline, 5 of the 43 individuals (11.6%) have HI antibodies > 40 to at least one of three A(H9N2) strains and 9 of them (20.9%) have detectable H9 protein binding antibodies (Supplementary Fig. 1b, c), although interestingly none were positive by both assays. None of the participants have HI antibodies to A(H7N9).

**Fig. 5:**
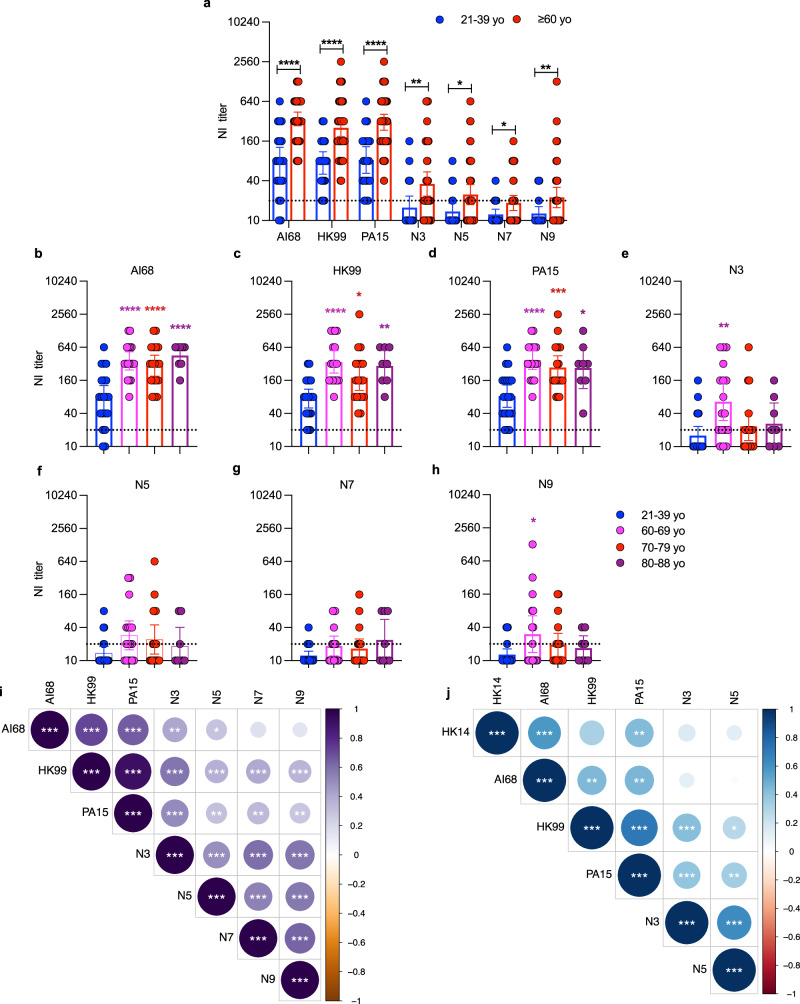
Antibody cross-reactivity to avian neuraminidase (NA) subtypes in the CARES samples. **a** Neuraminidase-inhibition (NI) antibody titers in older (≥60 yo, *n* = 43) and younger adults (21-39 yo, *n* = 20) to the N2 of A/Aichi/2/1968 (H3N2) (AI68) and avian N2s (from H9N2; HK99 and PA15), N3, N5, N7 and N9. Age-stratified NI-titers against **b** AI68, **c** HK99, **d** PA15, **e** N3, **f** N5, **g** N7 and **h** N9. Correlation of **i** baseline NI titers amongst the different NA subtypes and **j** post-infection NI-titer fold-change between reference infection strain A/Hong Kong/4801/2014 (H3N2) (HK14) with the different NA subtypes. NI-antibody was detected with ELLA using rgH6Nx viruses containing the NA genes of HK14, AI68, HK99, PA15, N3, N5, N7 and N9. Dotted lines indicate limits of detection. The bar graphs indicate the geometric mean antibody titer with 95% confidence intervals. Sample sizes; 60-69 (*n* = 17), 70-79 (*n* = 18) and 80-88 yo (*n* = 8). Statistical significance in (**a**) was analyzed using two-sided unpaired t-test (*p = 0.0102 for N5, *p = 0.0121 for N7, **p = 0.0052 for N3, **p = 0.0098 for N9, ****p < 0.0001 for AI68, HK99 or PA15). Statistical differences in **b**—**h** was compared to the 21-39 yo using two-sided one-way ANOVA, adjusted with Dunnett’s multiple comparison test. For **b**, ****p < 0.0001 for all age groups. For **c**, *p = 0.0139 for 70-79 yo, **p = 0.0023 for 80-88 yo, ****p < 0.0001 for 60-69 yo. For **d**, *p = 0.0114 for 80-88 yo, ***p = 0.0007 for 70-79 yo, ****p < 0.0001 for 60-69 yo. For **e**, **p = 0.0017 for 60-69 yo. For **h**, *p = 0.0328 for for 60-69 yo. Correlations in **i** and **j** were reported by Spearman’s correlation for each comparison using two-sided test and visualized using the ‘corrplot’ package (R version 0.92), and p-values were adjusted by controlling for the False Discovery Rate using the Benjamini-Hochberg method. In **i**; for AI68, -vs N3: **p = 0.003, -vs N5: *p = 0.017, - vs HK99 or PA15: ***p < 0.001; for HK99, -vs PA15, N3, N5, N7 or N9: ***p < 0.001, for PA15, -vs N5: **p = 0.001, -vs N7: **p = 0.001, -vs N9: **p = 0.007, -vs N3: ***p < 0.001; for N3, -vs N5, N7 or N9: ***p < 0.001; for N5, vs -N7 or N9: ***p < 0.001; for N7, -vs N9: ***p < 0.001. In **j**, for HK14, -vs PA15: **p = 0.010, -vs AI68: ***p < 0.001; for AI68, -vs HK99: **p = 0.010, -vs PA15, **p = 0.010; for HK99, -vs N5: *p = 0.015, -vs PA15 or N3: ***p < 0.001; for PA15, -vs N5: **p = 0.001, for N3: ***p < 0.001; -vs N5: ***p < 0.001.

To determine the extent of cross-reactivity, we aimed to generate rgH6Nx viruses bearing all nine avian NA subtypes, N1 to N9. However, only viruses bearing N5 (Group 1), N3, N7 or N9 (Group 2) were successfully rescued (Supplementary Table 7). Using baseline serum samples from CARES and data from the 21-39 yo individuals in Fig. 2 as comparators, we confirmed the presence of significantly higher NI titers to AI68 N2 and the two-representative avian N2s, HK99 and PA15 in older adults. We also found higher baseline titers against N3, N5, N7 and N9 compared to the younger adults (Fig. 5a). Because we have a larger sample size here compared to the Guangzhou serum samples, we were able to further stratify the responses into 60-69 (*n* = 17), 70-79 (*n* = 18) and 80-88 yo (*n* = 8) groups. The NI titers against AI68, HK99 and PA15 were mostly comparable for the three subgroups, although responses against HK99 were marginally higher in the 60-69 yo (Fig. 5b–d). N3 and N9 titers were also highest among 60-69 yo, with titers significantly higher compared to younger adults (Fig. 5e–h). No significant differences in NI titers were noted for N5 and N7 across the age groups (Fig. 5f, g). Interestingly, baseline AI68 N2 titers correlated strongly with HK99, PA15 and modestly with N3 and N5, but not with N7 or N9 (Fig. 5i). In contrast, baseline titers of HK99 correlated well with all avian NAs, suggesting that cross-reactivity driven by the AI68 N2 may be mostly limited to avian N2 and its closest phylogenetic neighbor, N3, whereas reactivity against HK99 will also likely include reactivity against broader avian NA subtypes.

In post-infection serum samples, 35 (81.4%) of the PCR-confirmed A(H3N2) cases showed seroconversion (four-fold increase in antibody titer) to the N2 of the representative circulating strain, A/Hong Kong/4801/2014 (H3N2) (HK14). Of this group, seroconversion rates were 39.5% to AI68, 18.6% to HK99, 25.6% to PA15, 18.6% to N3, 2.3% to N7, 4.7% to N9 and 11.6% to N5 (Table 2), displaying a similar trend in cross-reactivity as seen with the baseline sera. We asked if any correlations amongst the seroconversion events existed, i.e., whether seroconversion occurred against multiple strains or just single strains. We excluded N7 and N9 from the analysis due to low numbers of seroconversion events. There were significant positive correlations between seroconversion to HK14 N2 (circulating strain), AI68 and, curiously, PA15, but not HK99, N3 or N5 (Fig. 5j). However, seroconversions to the N2s of HK99 and PA15 were positively correlated to seroconversions to N3. Collectively, these data indicated that amongst the subtypes tested, avian NA cross-reactivity beyond N2 in baseline sera was more frequently observed in 60-69 yo, particularly against N3 and N9 in Group 2. Re-exposure to human N2 is likely to boost only AI68 N2, but can induce cross-group avian NA responses in some individuals that had pre-existing avian-type NA reactivity. Presence of cross-reactive H9 antibodies at baseline, as determined by either ELISA and HAI, did not seem to affect these NA responses as these differences and correlations still holds even when we restricted the analyzes to the 29 H9-seronegative (by both assays) individuals (Supplementary Fig. 4, Supplementary Table 8).

**Table 2:** Seroconversion rates to the different NA subtypes among the 43 A(H3N2)-infected participants in CARES cohort

NA Group	Group 1	Group 2						
Subtype	N5	Human N2		Avian N2		N3	N7	N9
Strain	GD08^b^	HK14^c^	AI68^d^	HK99^e^	PA15^f^	GD96^g^	SX10^h^	AH13^i^
Seroconversion^a^ No. (%)	5(11.6)	35(81.4)	17(39.5)	8(18.6)	11(25.6)	8(18.6)	1(2.3)	2(4.7)

^a^Seroconversion was defined as four-fold increase in neuraminidase inhibition (NI) antibody titers in paired serum samples.

^b^H6N5-A/duck/Guangdong/wy11/2008 (H5N5).

^c^H6N2_A/Hong Kong/4801/2014 (H3N2).

^d^H6N2_A/Aichi/2/1968 (H3N2).

^e^H6N2_A/Guineafowl/Hong Kong/WF10/99 (H9N2).

^f^H6N2_A/Pakistan/486/2015 (H9N2).

^g^H6N3_A/Duck/Guangdong/1/1996 (H7N3).

^h^H6N7_A/duck/Shanxi/3180/2010 (H10N7).

^i^H6N9_A/Anhui/1/2013 (H7N9).

### Role of HA-stalk and other HA-binding antibodies

HA-stalk antibodies have been reported to interfere with NI assays through steric hindrance^21^. To exclude the potential interference by HA-stalk antibodies in our data, we measured the level of antibodies specific for Group 1 and 2 HA-stalk regions using previously described constructs, pH1HA10-Foldon (designed from A/California/04/2009 (H1N1)) and HK68-H3-SI (designed from A/Hong Kong/1/1968 (H3N2))^22–24^ in the human and mice serum samples used in our study. Minimal antibodies were detected against both stalk proteins across all age groups in both the Guangzhou and CARES cohorts with no significant difference detected across any age groups (Supplementary Fig. 5a, b). We also tested the sera from the mouse immunization experiments for HA-stalk, H9 and H6 antibodies. As expected, since H6 belongs to Group 1, mice immunized with the rgH6Nx constructs elicited more Group 1 HA stalk antibodies compared to Group 2, although the titers were still relatively low. No significant difference of anti-HA stalk antibodies and anti-H9 binding were detected across the different groups of immunized mice (Supplementary Fig. 5c, d). No cross-reactivity were observed between H6 and H9 (Supplementary Fig. 5d). We conclude that HA-stalk and H9-binding antibodies are not major determinants of the cross-reactivity observed.

### Avian NA cross-reactivity profile stratified by year of birth

**Fig. 6:**
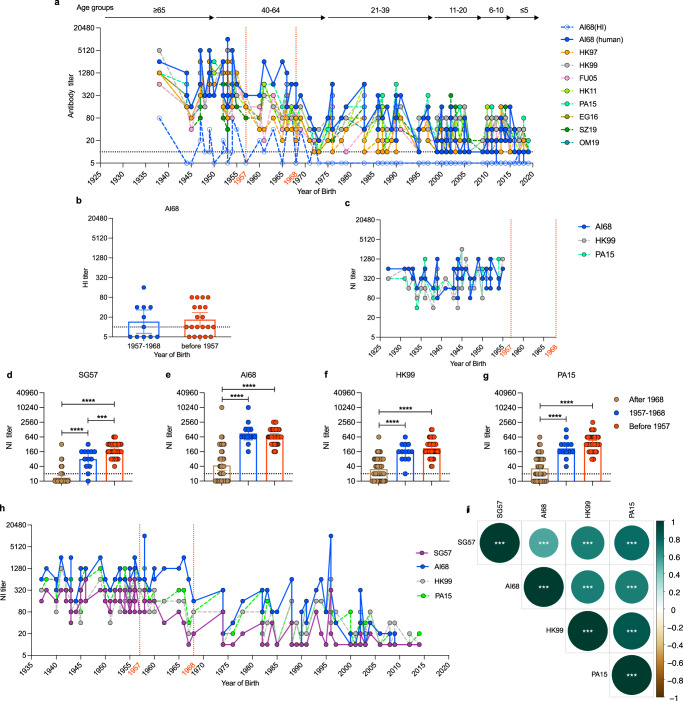
Antibodies cross-reactivity of neuraminidase between A/Aichi/2/1968 (H3N2), A/Singapore/1/1957 (H2N2) and avian A(H9N2). **a** The NI antibody profile against the NA of A/Aichi/2/1968 (H3N2) (AI68) and avian A(H9N2), and the HI antibody profile against AI68, as determined for Fig. 2, when stratified by year of birth. The age groups depicted in Fig. 2 are indicated on top of the graph. **b** HI-titer against AI68 in individuals born in between 1957 and 1968 (*n* = 11) and before 1957 (*n* = 20), as determined for Fig. 2. **c** The NI antibody profile against AI68 and avian A(H9N2) NA from CARES cohort samples, *n* = 43 individuals. **d**–**g** NI-titer against NA of A/Singapore/1/1957 (H2N2) (SG57), AI68 and two representative avian N2 from A/guinea fowl/Hong Kong/WF10/1999 (H9N2) (HK99), A/Pakistan/486/2015 (H9N2) (PA15) in the EPI-HK cohort. Analyzes were stratified to individuals after 1968 (*n* = 47), between 1957 to 1968 (*n* = 13), and individuals born before 1957 (*n* = 45). **h** The NI antibody profile of the EPI-HK participants against human N2; SG57 and AI68, and avian N2; HK99 and PA15, when stratified by year of birth. **i** Correlation of NI titers between SG57 N2 and the different neuraminidase subtypes in *n* = 58 individuals born in or before 1968 from EPI-HK cohort. Dashed lines indicate the 1957 A(H2N2) and 1968 A(H3N2) pandemics. Dotted lines indicate limits of detection. The bar graphs indicate the geometric mean antibody titer with 95% confidence intervals. Statistical significance in **b** was analyzed using two-sided unpaired t-test. Statistical significance between different age groups in **d**–**g** was analyzed using two-sided one-way ANOVA, adjusted with Tukey’s multiple comparisons test (***p = 0.0008 for 1957-1968 vs before 1957, ****p < 0.0001 for after 1968 vs 1957-1968 or 1968 vs before 1957 in (**d**); ****p < 0.0001 for after 1968 vs 1957-1968 or 1968 vs before 1957 in **e**, **f** or **g**). Correlation in **i** was reported by Spearman’s correlation for each comparison using two-sided test and visualized using the ‘corrplot’ package (R version 0.92), and p-values were adjusted by controlling for the False Discovery Rate using the Benjamini-Hochberg method (***p < 0.001 for each pairwise comparison).

When we parsed the N2 cross-reactive data in the Guangzhou cohort (Fig. 2d) according to year of birth, we observed distinct reactivity profiles in relation to the AI68 N2 titers. Individuals born between 1957 to 1968 (52-63 yo) showed high AI68 N2 titers but modest reactivity to avian N2 strains (Fig. 6a). In contrast, individuals born prior to 1957, showed high AI68 and avian N2 titers. HI titers to AI68 were detected in most of these individuals, with no difference in the average GMT between individuals born before 1957 with those born between 1957 to 1968 (Fig. 6b). This suggests that the cross-protection conferred by the pooled sera from 40-64 yo against ZJ19 in Fig. 2g likely came from these individuals with high AI68 N2 antibody titers. Individuals in the CARES cohort, who were all born before 1955, demonstrated a similar high avian N2 cross-reactivity profile (Fig. 6c). No year-of-birth pattern was observed for reactivity against the other avian NA subtypes in the CARES samples (Supplementary Fig. 6). Collectively, this data suggests individuals born prior to 1957 had higher avian N2 cross-reactivity profile compared to those born between 1957 and 1968.

To evaluate whether the high avian N2 cross-reactivity was related to the 1957 pandemic exposure, we tested 112 age-stratified serum samples collected from a separate community-based cohort study on respiratory virus infections in Hong Kong, “Evaluating Population Immunity in Hong Kong” (EPI-HK)^25^. After excluding 7 individuals with positive H9 HI titers against HK11 and SZ19, we tested the remaining 105 samples for antibodies against N2 from A/Singapore/1/1957(H2N2) (SG57), AI68 and the two-representative avian N2, HK99 and PA15. Results from this cohort showed that N2 titers to SG57 were highest in those born before 1957 and gradually declined thereafter (Fig. 6d). In contrast, N2 titers to AI68 were not different between those born before 1957 and those born between 1957 and 1968 (Fig. 6e). This birth-year response pattern was consistent to that of HK99 and PA15 although the SG57 titers were generally lower compared to the two avian strains and AI68 (Fig. 6f–h). The N2 titers were well-correlated amongst the tested strains but a stronger correlation was noted between the avian N2s with SG57 than AI68 (Fig. 6i). Given that the AI68 N2 still shares antigenic similarity with the 1957 and avian N2, it is likely that exposure to the 1968 N2 elicited antibodies that can cross-react with avian N2 in naïve population while boosting the pre-existing N2 antibodies, resulting in higher avian N2 titers in the pre-1957 cohort.

## Discussion

Our study investigated the cross-reactive and protective potential of NA antibodies to A(H9N2) AIVs. We discovered that older adults exhibited high titers of cross-reactive NA antibodies against various A(H9N2) viruses, that is likely conferred by exposures to the 1957 and 1968 pandemic viruses. Our mouse studies indicated that these cross-reactive antibodies are protective and data from the CARES cohort suggest that they can still be recalled after seasonal A(H3N2) infection in some individuals.

The NA cross-reactivity profile outlined in Fig. 6 reveals three intriguing birth-year specific patterns; (i) high antibody reactivity to SG57, AI68 and avian N2 in those born prior to 1957, (ii) high AI68 but moderate SG57 and avian N2 reactivity in those born between 1957 and 1968 and (iii) declining AI68, and low SG57 and avian N2 reactivity in those born after 1968. Based on modeling estimates^26^ and seroprevalence studies^27^, the first exposure to influenza typically occur within the first decade of life^15-16,^ resulting in peak titers to strains circulating within this period (Fig. 2b). With this expectation, those born prior to 1957 would be exposed to a 1957 avian origin-N2, while those born within a decade prior to 1968 would be exposed to the 1968 N2 that have drifted from its 1957 N2-avian predecessor and adapted in humans^28,29^. Those born after 1968 would be exposed to further human-adapted progenies of AI68 N2, conferring more human-like antigenic specificities. This explanation is also consistent with our HI titer profile to AI68 and the report by E. te Beest et al., where they observed peak IgG titers to AI68 in Dutch adults born between the years of 1954 to 1969^30^. Because of antigenic similarities between the 1957 and 1968 N2, the high avian NA cross-reactivity profile in those born prior to 1957 could also be the result of boosting during re-exposure to 1968 N2. Because the N9 and N3 titers correlated with avian NA titers (Fig. 5i) and was observed only within this narrow age range, it may be related to avian-type NA reactivity, potentially conferred by exposure to the H2N2 pandemic, although this needs to be tested experimentally.

Intriguingly, early A(H9N2) vaccine trial reported by Stephenson et al., identified high pre-existing H9 HI-antibodies in individuals born prior to 1968^31^. The authors concluded this is likely due to cross-reactivity between H2 and H9, as they did not detect significant pre-existing N2 antibodies by microneutralization assay. However, this conclusion could be confounded by the observation that NA antibodies do not readily neutralize viruses in vitro^32,33^ and that certain avian neuraminidases possess hemagglutination ability^33–35^. Hence it is possible that these high-preexisting H9 HI-titers were due to NA-antibodies. Furthermore, we did not detect significant levels of H9-binding antibodies in both the Guangzhou and CARES cohort that is consistent with the protection observed in mice challenge experiments, and our passive transfer experiments clearly demonstrate a protective role for N2 antibodies against A(H9N2) challenge.

We also could not exclude the possibility that an avian N2 predecessor may have circulated in China prior to its emergence in 1957. Alternatively, another hypothesis to explain the high reactivity to avian NA in individuals ≥65 yo is the contribution of N1 circulating prior to 1957. The N1 that emerged during the 1918 pandemic is postulated to have also originated from avian reservoirs^36,37^. The higher avian N2 titers observed in this age group could have been compounded by exposure to this N1, although one would expect this NA to have lost its avian-like antigenicity after 20 years of human circulation. Due to the scarcity of data, further study will be required to prove either hypothesis.

Data from our mouse studies suggest that exposure to 1968-like N2s protected against A(H9N2) challenge, which would explain why sera from 40-64 yo were protective against A(H9N2) challenge in the passive transfer experiments. This age group included individuals that showed the highest AI68 N2 antibody titers despite low avian N2 reactivity. Post-infection serum samples from the CARES cohort suggest that these cross-reactive N2 antibodies can be recalled even by a contemporary A(H3N2) virus in some individuals in this group, suggesting that cross-reactive memory B cells persisted and could be activated upon re-exposure^38^. Data in Fig. 5j showed that seroconversion to HK14 correlated with PA15, but not HK99, which suggests that either PA15 may share some epitopes with HK14 or the PA15 N2 may have more accessible epitopes, lending to higher sensitivity in antibody detection with the assay. The presence of these cross-reactive memory B cells suggests protective capacity against AIVs bearing N2 or potentially even N3 or N5. Furthermore, because the CARES cohort were sampled at six-month intervals, these post-infection, cross-reactive NA antibody responses are likely stable and of high affinity, rather than a transient polyreactive response. However, despite the high baseline titers, this capacity does not appear to extend too broadly across the two groups since N5, N7 and N9 recall responses after infection are minimal, which could explain why H7N9 was still able to cause spillover infections in predominantly older adults during its early emergence in China in 2013^39^.

There are some limitations to our study. The avian N2 strains evaluated in the serosurvey were mainly Eurasian lineages and did not include North American lineages. However, unlike the Eurasian lineage A(H9N2) viruses, the North American A(H9N2) viruses have not caused any major poultry outbreaks or been associated with human infections in recent years. As such, the focus of our study was to evaluate cross-reactivity against A(H9N2) variants of public health concern. Nonetheless, a comprehensive characterization of the breadth of N2, or even other avian NA subtypes, antibody cross-reactivity present in the human population may reveal important insights into the antigenic relationships of ancestral human N2 viruses with avian predecessors. A larger sample size could further clarify the patterns of cross-reactivity. In addition, it is unknown how representative the age-specific profile of NA cross-reactivity described here would compare across different geographic locations, although we hypothesize that it will likely be generalizable based on our observation that exposure to AI68 alone was sufficient to confer cross-reactive antibodies and protection against A(H9N2) challenge. As most A(H9N2) viruses do not cause significant morbidity in mice^40^, we were only able to assess protection using this particular isolate of A(H9N2), ZJ19, which showed measurable morbidity at a dose of 10^7^ PFU. We were still able to demonstrate protection conferred by the human or mouse sera, despite the relatively high dose used. Lastly, pooled human sera may contain protective non-NA antibodies. We attempted to address this by purifying NA antibodies but were unsuccessful in recovering sufficient antibodies to perform the passive transfer experiments. Whilst we cannot exclude the contributions of non-NA antibodies, we suspect that their contribution was minimal based on our observation that priming with rgH6Nx viruses did not provide protection against A(H9N2) challenge, despite both being Group 1 AIVs.

To summarize, we showed that cross-reactive and protective avian N2 antibodies are present in the population in an age-dependent manner due to past exposure to avian-like NAs during the 1957 and 1968 pandemics. This is likely a major determinant of age-specific morbidity associated with A(H9N2) AIV infection and should be further explored within the context of other zoonotic influenza viruses. Likewise, NA-immunity should be considered in risk-assessment evaluations of zoonotic influenza viruses.

## Methods

### Ethics statement

Studies using samples of the Guangzhou cohort received ethical approval from the Institutional Review Board of the First Affiliated Hospital of Guangzhou Medical University (Ref: ES-2023- K011-01). Written informed consent was waived as de-identified residual sera were used. The CARES study received ethical approval from the Ethics Committee of Jiangsu Provincial Center for Disease Prevention and Control (Ref: JSJK2015-B013-02) while the EPI-HK study received ethical approval from the Institutional Review Board of the University of Hong Kong (Ref: UW15-404 and UW19-720). All participants or their guardians in the CARES and EPI-HK study provided informed written consent.

### Phylogenetic analysis and strains selection

NA from A(H3N2) vaccine strains were selected as representative strains, while AIV strains were selected based on prototypical strains of geographical and temporal importance. Viral genomes were downloaded from the Influenza Research Database (http://www.fludb.org) and the Global Initiative on Sharing All Influenza Data (https://gisaid.org). Maximum likelihood phylogenetic trees were inferred using the maximum likelihood method with the GTR + G + I nucleotide substitution model in MEGA-X^41^. Branch support was estimated using 1000 bootstrap replicates. Phylogenetic relationships and sequence similarity were used to select representative A(H3N2) and A(H9N2) strains for serological studies. These included prototypical A(H9N2) viruses representing BJ94 subclades, A/chicken/Hong Kong/G9/1997 (HK97) and A/chicken/Fujian/9290/2005 (FU05)^42^, as well as a BJ94/F98 A(H9N2) virus, A/Suzhou/GIRD01/2019 (SZ19), which was isolated from a critical case of respiratory illness in a 9-year-old^43^. We also included the G1 subclade A(H9N2) viruses A/guinea fowl/Hong Kong/WF10/1999 (HK99), A/chicken/Hong Kong/YU250W/2011 (HK11), A/Pakistan/486/2015 (PA15), A/chicken/Egypt /S12568C/2016 (EG16), and A/Oman/2747/2019 (OM19). Notably, PA15, EG16 and OM19 have recently been identified as candidate vaccine viruses (CVVs) for pandemic preparedness (https://www.who.int/publications/m/item/a(h9n2)—northern-hemisphere-2022-2023).

### Human cohorts

To determine the antibody seroprevalence to human and avian N2 in the human population, we used residual human serum samples collected by the Department of Laboratory Medicine of the First Affiliated Hospital of Guangzhou Medical University in Guangzhou, China and serum collected from a community-based longitudinal cohort study on respiratory virus infections in Hong Kong, “Evaluating Population Immunity in Hong Kong” (EPI-HK)^25^. Serum samples from the Guangzhou cohort were collected during physical examinations generally conducted once a year for education and employment purposes, whereas the EPI-HK baseline serum samples were collected upon enrollment in July 2020.

In the Guangzhou cohort, twenty serum samples per age group were collected based on previous findings that predicted probability of exposure^24^; i.e ≤5yo: no or at most 1 exposure, 6-10 yo: at least 1 exposure, 11-20 yo: likely more than 1 exposure, 21-39 yo: multiple exposures. For the 40-64 yo and the > 65 yo, we further considered the potential imprint profile due to early life exposure based on the viruses in circulation at time of birth (Supplementary Table 2). In the EPI-HK cohort, we modified the age groups to 0–10, 11–20, 21–39, 40–64, 65–74 and > 75 years old to prioritize the older adults. Both cohorts were pre-specified to include equal ratios of males to females. HI assays were conducted to identify and exclude individuals that may have been exposed to subtype A(H9N2) IAVs and to confirm those with past exposures to subtype A(H3N2) IAVs.

To determine whether the antibody reactivity against NA protein can also be observed in other avian NA subtypes and if these antibody responses could be recalled after seasonal influenza virus infection, we use paired baseline and post-infection serum samples from 43 PCR-confirmed cases of A(H3N2) influenza virus infections identified from the China Ageing REspiratory infections Study (CARES). CARES was a longitudinal surveillance cohort for respiratory viral infections, conducted amongst community-dwelling adults between 60–89 years of age at enrollment during the winter of 2015-2017 in Suzhou and Yancheng, Jiangsu province, China^20^.

### Virus generation, propagation and titration

Wild type (wt) and reverse genetics (rg) viruses were propagated in 10-day-old embryonated chicken eggs. To generate rg-viruses, genes of interests were synthesized (Supplementary Table 7) (Sangon Biotech Co., Ltd.), cloned into pHW2000 and used to rescue viruses based on the eight plasmid rg-system^44^. Briefly, plasmids encoding the HA and NA genes of interests were co-transfected along with the six internal genes were of A/Puerto Rico/8/1934 (H1N1) into 293 T cells at 1 µg each using Lipofectamine 2000 (Invitrogen). Wild type A/chicken/Zhejiang/198/2019 (H9N2) (ZJ19) virus and A/Aichi/2/1968 (H3N2) were used for the challenge experiment. rgH3N2 viruses were generated for HI assay, whereas rgH6Nx viruses were generated for ELLA. The rgH6Nx viruses were generated using the mismatched H6 from A/teal/Hong Kong/W312/1997 (H6N1). Sanger sequencing was used to confirm virus genome sequences. Virus stocks were stored at −80 °C and titrated by plaque assay in Madin Darby Canine Kidney (MDCK) cells that were maintained in minimal essential medium (MEM) supplemented with 10% fetal bovine serum (Thermo Fisher Scientific).

### Hemagglutination inhibition assay

Hemagglutinin inhibition (HI) assays were performed according to standard protocol (https://apps.who.int/iris/handle/10665/44518). Briefly, sera were pre-treated with receptor-destroying enzyme (RDE, Denka Seiken) to remove non-specific inhibitors of agglutination and heat-inactivated at 56 °C for 1 h. Serum samples were titrated in serial two-fold dilutions with a starting dilution of 1:10 and incubated with four agglutinating doses of test antigens for 45 min. Antibody titers were measured as the reciprocal of the highest dilution causing complete hemagglutination inhibition of 1% guinea pig red blood cells (Guangzhou Ruite Biotechnology Co., Ltd.).

### Neuraminidase inhibition (NI) assay

Functional NA antibodies were detected by enzyme-linked lectin assay (ELLA) using rg viruses expressing the NA of the target strains and a mismatched HA. Microtiter plates were coated overnight with fetuin (BIO-RAD) prior to the addition of two-fold serial dilutions of heat-inactivated sera starting with a dilution of 1:20 and a pre-determined concentration of recombinant virus, incubated for 18 h at 37 °C. Peroxidase-labeled peanut agglutinin (lectin) (Sigma) was added to the reaction and incubated for 2 h at room temperature. Bound lectins were detected with 3,3,5,5-Tetramethylbenzidine (TMB) Liquid Substrate System (Life Technologies). NI titers were measured as the reciprocal of the highest dilution that resulted in more than 50% signal inhibition compared to virus-only wells. Paired sera were tested together in the same run. A serum with previously pre-defined titer against the reference antigens was used as an internal control.

### Enzyme-linked immunosorbent assay (ELISA)

Recombinant full-length HA proteins (Sinobiologicals) or the HA-stalk proteins were coated at 0.5 mg/ml onto 96-well high binding immunoassay plates in coating buffer overnight at 4 °C. Negative wells were coated with buffer only. Plates were washed three times with phosphate buffered saline (PBS) supplemented with 0.05% Tween-20 (PBS-T) and blocked for two hours with PBS supplemented with 5% fetal bovine serum (Lonsera). After removing the blocking solution, 50 μl of serially diluted sera was added at a starting dilution of 1:200 in duplicate wells and incubated for two hours at 37 °C. Sera were then removed and plates were washed three times with PBS-T. 50 μl/well anti-human (Bioss, bs-0297G-HRP) or anti-mouse IgG (Bioss, bs-0296G-HRP) secondary antibody, diluted 1:20,000 or 1:10,000 in 5% FBS-PBS-T, was added to each well and incubated for 1 h at 37 °C. Secondary antibody was then removed and the plates were washed three times with 220 μl/well PBS-T, and 50 μl/well of TMB was added. After 15 min, 50 μl/well of 0.5 M H_2_SO_4_ was added to stop the reaction and the absorbance was read at 450 nm. End-point titer was calculated as the reciprocal dilution that gave a positive/negative optical density readout ratio of >2. Sera from H1 or H3 stalk-immunized mice was used as a positive control.

### Mouse experiments

All mouse experiments were conducted in accordance with institutional animal care guidelines and were approved by the Animal Care Committee of Guangzhou Medical University. We used female mice in this study as they develop more robust antibody IgG responses compared to male mice^45^. Mice were purchased from Zhejiang Vital River Laboratory Animal Technology Co., Ltd and housed in the Experimental Animal Center of Guangzhou Medical University under in a ventilated isocage, in a room with a 12- h light/dark cycle. The temperature and humidity are maintained at 20–26 °C with 30%–70% percent humidity. Food and water were provided *ad libitum*. Infection was performed by intranasal inoculation of 30 μl virus inoculum after isoflurane anesthesia. Mice were monitored daily for weight loss and signs of disease. Animals that reached humane endpoints, such as severe symptomology or >30% weight loss, were euthanized. To determine lung viral loads, lungs were weighed, homogenized using a TissueLyser II (QIAGEN) and pelleted by centrifugation. The collected supernatant was titrated in MDCK cells. Experimental schema of mouse experiments was created with BioRender.com (https://www.biorender.com).

### Human sera passive transfer-challenge mouse experiment

6-8 weeks old female Balb/c mice (*n*  =  8 to 10 per group, depending on the available volume of pooled sera) were intraperitoneally injected with 350 μl of pooled human sera (pooled in equal volume from all samples) from the respective age groups and challenged 18 hours later with 10^7.1^ PFU of ZJ19. Animals were monitored daily for signs of discomfort and weight loss for up to 14 days post inoculation (DPI).

### Prime-challenge mouse experiment

In the first prime-challenge experiment, groups of 6-8 weeks old female Balb/c mice (*n* = 11/group) were inoculated with either phosphate-buffered saline (PBS) or the following priming viruses: wild-type H3N2-AI68 (A/Aichi/2/1968) virus, and reassortant rgH3N2-SG16, containing the HA and NA genes of A/Singapore/INFIMH160019/2016 (H3N2), or rgH1N1-MI15 containing the HA and NA genes of A/Michigan/45/2015 (H1N1) at 10^2^, 10^6^, 10^2^ PFU respectively. In the second prime-challenge experiment, the rgH6Nx viruses; rgH6N2-AI68, rgH6N2-SG16 or rgH6N1-MI15 containing the NA genes of AI68, SG16 or MI15, the HA gene of A/teal/Hong Kong/W312/1997 (H6N1) and remaining genes of A/Puerto Rico/8/1934 (H1N1) were used at 10^3^, 10^4^ or 10^3^ PFU, respectively. The positive and negative control groups were inoculated with 10^5.5^ PFU of A/chicken/Zhejiang/198/2019 (H9N2) (ZJ19) or PBS. Mice were boosted again with the same viruses at 21DPI and challenged with 10^7.1^ PFU of ZJ19 at 51 DPI. After challenge, lungs were collected at 3 and 6 DPI for virus titration (*n* = 3) and the remaining mice (*n* = 5) were monitored for 14 DPI to study disease pathogenesis. Sera were collected at baseline, at secondary boost and after viral challenge. Virus titers were titrated by tissue culture infectious dose 50% (TCID50) assay in MDCK cells.

### Immune sera passive transfer-challenge mouse experiment

To assess the cross-protective efficacy of NA antibodies, sera were collected from 6-8 weeks old female Balb/c mice (*n* = 14/group) after two doses of inoculation with rgH6Nx viruses rgH6N2-AI68, rgH6N2-SG16 or rgH6N1-MI15 at 10^3^, 10^4^, and 10^3^ PFU, respectively. Positive control serum was pooled from mice inoculated with 10^5.5^ PFU of ZJ19 and negative control serum was pooled from PBS-inoculated mice. 350 μl of pooled, heat-inactivated mouse immune sera were passively transferred by intraperitoneal injection (*n*  =  8 to 10 per group, depending on the available volume of pooled sera). Recipient mice were challenged 18 h later with 10^7.1^ PFU of ZJ19 and monitored for weight loss and symptoms for up to 14 DPI^46^.

### Statistical analysis

Statistical analyses were performed using GraphPad Prism (version 9), while correlations amongst different strains were performed by using corrplot package (version 0.92) (https://github.com/taiyun/corrplot) and Complex Heatmap was generated by using Complex Heatmap package (version 2.13.1) (http://bioconductor.org/packages/release/BiocViews.html#___Software)^47^ using R software (version 4.2.2). For antibody data, samples that did not meet the assay detection threshold were assigned a value half of the starting dilutions, i.e., mouse sera with no detectable signal in ELLA and HI assays were assigned a titer of 5 and samples of human sera with no detectable signal in ELLA assays were assigned a titer of 10. Antibody data were log transformed for statistical testing. Point estimates were expressed as geometric mean titers (GMT) with 95% confidence intervals (CI) and fold-changes were expressed as geometric mean fold changes (GMFCs) with 95% CI. Statistical significance between paired samples were determined using paired-tests while differences between groups were determined by unpaired t-tests or one-way ANOVA. Mouse survival was analyzed using Gehan-Breslow-Wilcoxon test; *p* < 0.05 was considered significant.

### Reporting summary

Further information on research design is available in the Nature Portfolio Reporting Summary linked to this article.

### Supplementary information

Supplementary Information

Peer Review File

Reporting Summary

## Data Availability

The data generated in this study are provided in the Supplementary Information/Source Data file. Source data supporting the findings of this study are available on figshare here.
