# Supplementary material for: A(H2N2) and A(H3N2) influenza pandemics elicited durable cross-reactive and protective antibodies against avian N2 neuraminidases
Source: Nat Commun. 2024 Jul 3;15:5593. doi: 10.1038/s41467-024-49884-9 (PMC11222539; doi:10.1038/s41467-024-49884-9)
Supplement: Supplementary file 1 — Supplementary Information [file 41467_2024_49884_MOESM1_ESM.pdf]

Supplementary Material

A(H2N2) and A(H3N2) influenza pandemics elicited durable cross-reactive and protective antibodies against avian N2 neuraminidases

Liang et al., 2024

Supplementary Fig.1 | H9 antibody profile in the Guangzhou and CARES cohorts individuals as assayed using IgG-Enzyme-linked immunosorbent assay (ELISA). (a) IgG-titer to H9 protein of A/Hong Kong/1073/99 (H9N2) in the Guangzhou cohort. (b) Hemagglutination-inhibition (HI) titer against three A(H9N2) strains; A/chicken/Hong Kong/YU250W/2011 (HK11), A/guinea fowl/Hong Kong/WF10/1999 (H9N2) (HK99) and A/Suzhou/GIRD01/2019 (SZ19) in CARES cohort. HI-assay was performed with rgH9N2 viruses containing the HA and NA genes in the backbone of A/Puerto Rico/8/1934 (H1N1) (PR8). (c) IgG-titer to H9 protein of A/Hong Kong/1073/99 (H9N2) in CARES cohort by ELISA. Dotted lines in (a)-(c) indicate limits of detection. Antibody data were expressed as geometric mean titers ± 95% confidence intervals. Statistical significance in (a) or (c) was determined using two-sided test by one-way ANOVA using Tukey's multiple comparisons test (For (a), **p=0.0078 for ≤0-5 yo group vs ≥65 yo group, **p=0.0078 for 11-20 yo group vs ≥65 yo group). Sample sizes, for Guangzhou cohort (N=70): n=10 each in ≤0-5, 6-10, 11-20 and 21-39 yo group, n=12 in 40-64 yo group, and n=18 for ≥65 yo group. For CARES (N=43), n=17 in the 60-69 yo group, n=18 in the 70-79 yo group and n=8 in 80-88 yo group.

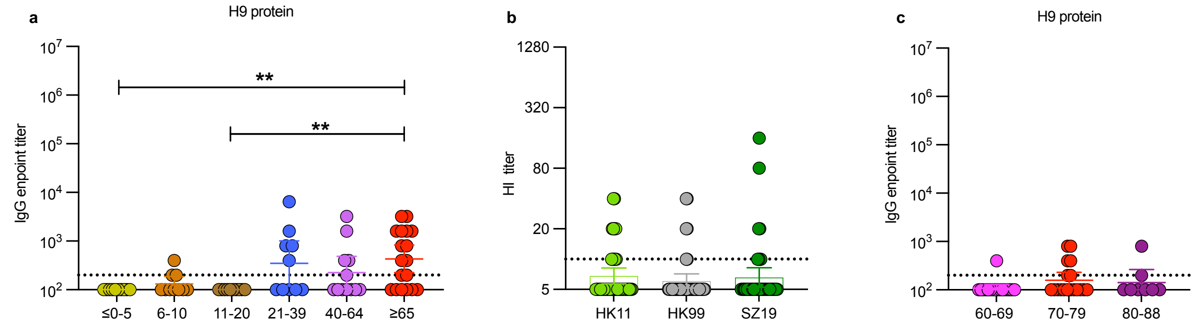

Supplementary Fig.2 | NA antibodies against human N2 and avian N2 in anti-H9 binding negative participants from Guangzhou cohort. Age-stratified neuraminidase-inhibition (NI) antibody titers to (a) selected human A(H3N2) or (b) A (H9N2) AIV. NI was performed with rgH6Nx viruses containing the containing the NA gene of the selected A(H3N2) or A(H9N2) viruses. Dotted lines in (a), (b) indicate limits of detection. Data expressed as geometric mean titer with 95% confidence intervals. Sample sizes, n=10 in ≤0-5 yo group, n=7 in 6-10 yo group, n=10 in 11-20 yo group, n=5 in 21-39 yo group, n=7 in 40-64 yo group, and n=6 for ≥65 yo group.

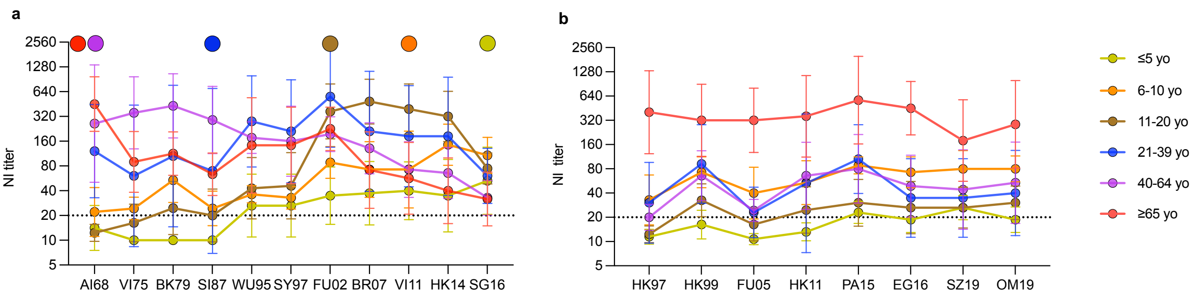

Supplementary Fig.3 | Protective efficacy of prime-challenge vaccination regimens against challenge with subtype A(H9N2) influenza virus in mice. Weight loss (a) and (d) and survival (b) and (e) of mice previously immunized with; (a, b) a wild-type (wt) A/Aichi/2/1968 (H3N2) (AI68), rg-derived A/Singapore/INFIMH160019/2016 (H3N2) (SG16) and rg-A/Michigan/45/2015 (H1N1) (MI15) or (d,e) recombinant H6Nx viruses bearing the NA from AI68, SG16 and MI15. Positive and negative control groups are immunized with wt-A/chicken/Zhejiang/198/2019 (H9N2) (ZJ19) and PBS, respectively. (c) and (f) Viral titers in lungs of mice in different immunization groups were determined on days 3 and days 6 post inoculation with H9N2-ZJ19 virus. Weight loss in (a) and (d) were expressed as mean ± standard deviation, survival in (b) and (e) were analyzed using two-sided Gehan-Breslow-Wilcoxon test, with the H3N2-AI68 or rgH6N2-AI68 group as reference. Each group had n=5 mice, except for rgH1N1-MI15 group which had 4 mice. Viral titers in lung were expressed as mean with SD, with n=3 mice per group. Statistical significance in (c) and (f) was compared to H3N2-AI68 or rgH6N2-AI68 and was analyzed using two-sided one-way ANOVA test, corrected for multiple comparisons using Dunnett's test (For (c), **p=0.0012 for PBS, ****p<0.0001 for H9N2-ZJ19; for (f), ****p<0.0001 for H9N2-ZJ19).

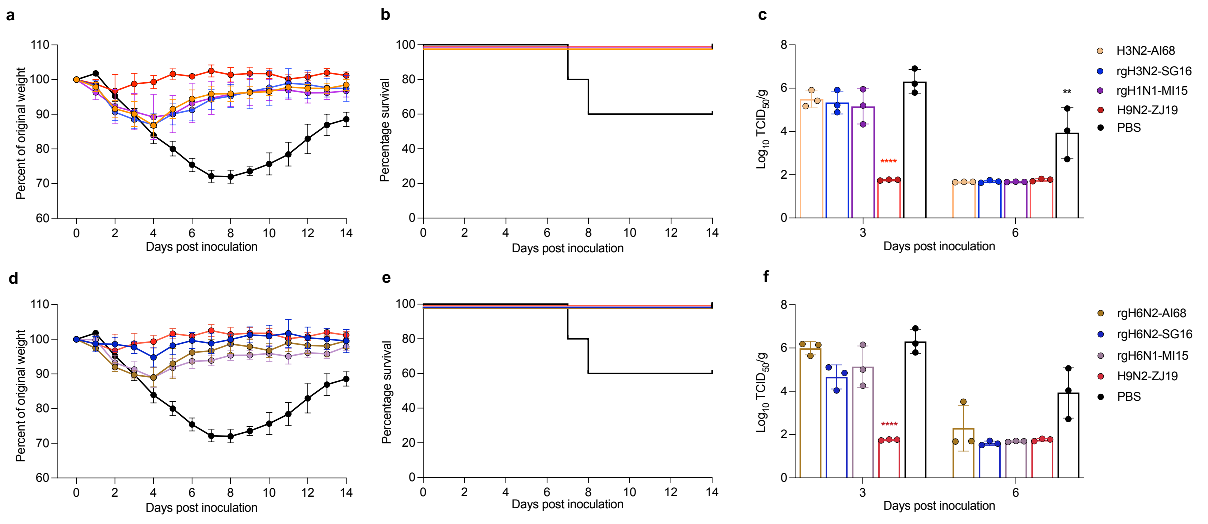

Supplementary Fig.4 | NA antibody responses against different neuraminidase subtypes amongst H9-negative individuals from CARES cohort. (a) Neuraminidase-inhibition (NI) antibodies profiles against different neuraminidase subtypes as determined in Fig.5 after excluding 14 H9-positive individuals in the CARES cohort, (b)-(h) age-stratified NI-antibody titers, (i) Correlation of baseline NI antibody titers and (j) antibody fold-change post-infection. Dotted lines in (a) indicate limits of detection. Antibody data were expressed as geometric mean titers ± 95% confidence intervals. Sample sizes, n=20 in the 21-39 yo group (from Guangzhou cohort); n=14 in the 60-69 yo, n=10 in the 70-79 yo group and n=5 in 80-88 yo group. Statistical significance in (a) was analyzed using two-sided unpaired t-test (*p=0.0173 for N5, *p=0.0266 for N7, *p=0.0184 for N9, **p =0.0044 for N3, ***p=0.0001 for HK99, ****p<0.0001 for AI68 or PA15). Statistical significance in (b)-(h) was determined using two-sided one-way ANOVA, with the 21-39 yo group as reference and adjusted with Dunnett's multiple comparisons test. For (b), **p=0.0022 for 70-80 yo, **p=0.0067 for 80-88 yo, ***p=0.0001 for 60-70 yo; for (c), ****p<0.0001 for for 60-70 yo; for (d), *p= 0.0372 for 70-80 yo, ****p<0.0001 for 60-70 yo; for (e), **p= 0.0018 for 60-70 yo; for (h), *p=0.0468 for 60-70 yo). Correlation in (i) and (j) was reported by Spearman's correlation for each comparison using two-sided test, with p-values adjusted by controlling for the False Discovery Rate using the Benjamini-Hochberg method. In (i), for AI68, - vs N3: **p=0.008, -vs N5: *p=0.011, -vs HK99 or PA15: ***p <0.001; for HK99, -vs N9: **p=0.002, -vs PA15, N3, N5 or N7: ***p <0.001; for PA15, - vs N7: **p=0.002, -vs N9: **p=0.005, -vs N3 or N5: ***p <0.001; for N3, -vs N5: **p=0.001, -vs N7 or N9: ***p <0.001; for N5, -vs N7 or N9: ***p <0.001; for N7 vs N9: ***p <0.001. In (j), for HK14 vs AI68: ***p <0.001; for AI68, -vs HK99: **p=0.010, -vs PA15: *p=0.031; for HK99, -vs PA15: ***p <0.001, -vs N3: **p=0.009; for PA15, -vs N5: **p=0.004, -vs N3: ***p <0.001; for N3 vs N5***p <0.001.

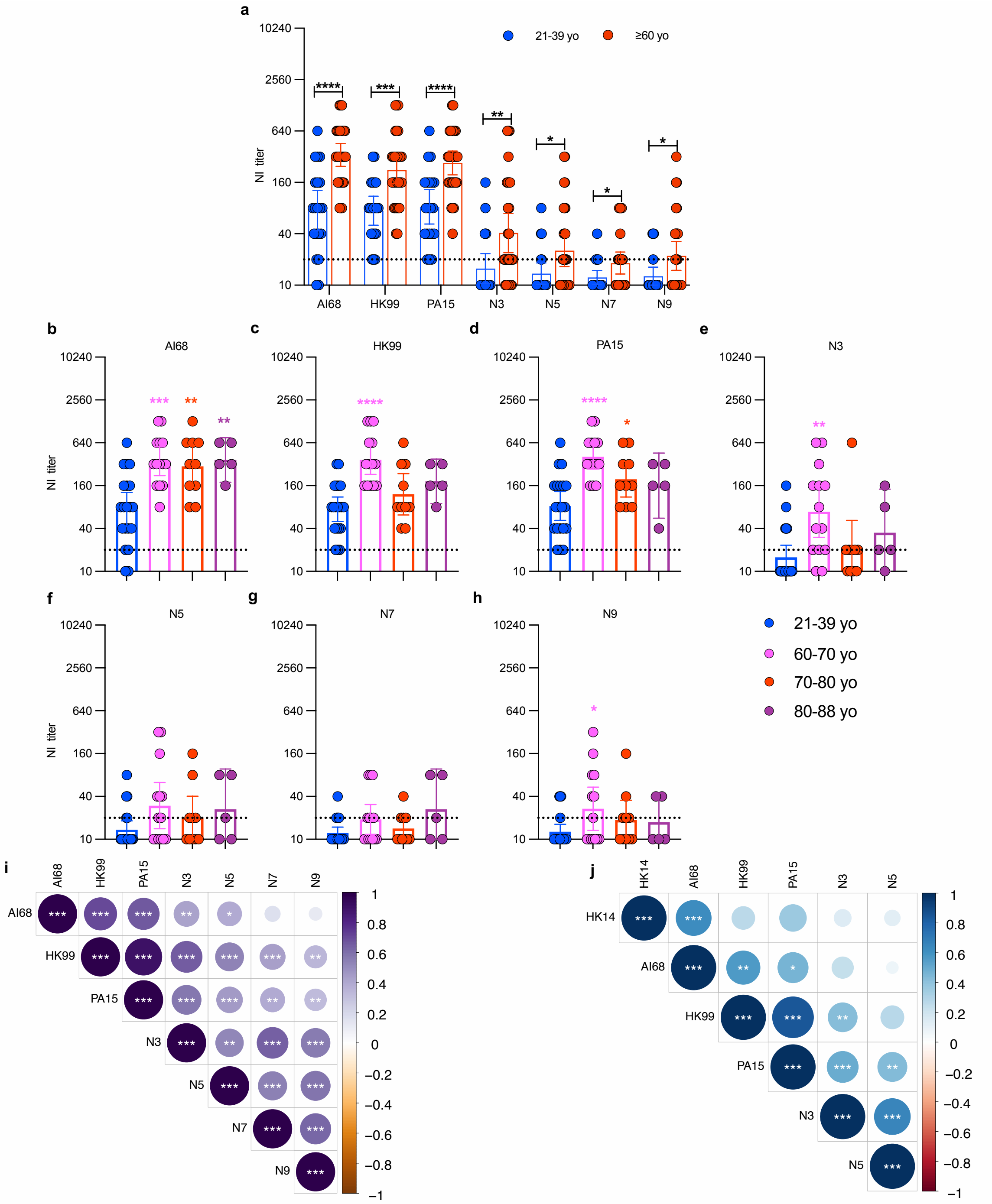
a
b
c
d
e
f
g
h
i
j
NI titer
21-39 yo
≥60 yo
60-70 yo
70-80 yo
80-88 yo
AI68
HK99
PA15
N3
N5
N7
N9
HK14

Supplementary Fig.5 | Role of HA-stalk and other HA-binding antibodies. HA stalk antibody profile of individuals from (a) Guangzhou or (b) CARES cohort in the different age groups. Sera of mice immunized with H1 stalk of pH1HA10-Foldon (designed from A/California/04/2009 (H1N1)) or H3 stalk of HK68-H3-SI (designed from A/Hong Kong/1/1968 (H3N2)) were included as control. (c) HA stalk antibody profile or (d) anti-H9 protein of A/Hong Kong/1073/99 (H9N2) and anti-H6 protein of A/chicken/Hong Kong/17/77(H6N4) antibodies in immunized mice sera from passive transfer-challenge mouse experiment. Dotted lines in (a)-(d) indicate limits of detection. Binding antibody was detected using IgG-Enzyme-linked immunosorbent assay (ELISA). Bar graphs indicate the geometric mean titers with 95% confidence intervals, difference in titers in (a)-(d) was determined using two-sided test by one-way ANOVA, adjusted with Tukey's multiple comparisons test (For (d), *p=0.0425 for rgH6N2-AI68 vs H9N2-ZJ19, *p=0.0425 for rgH6N1-MI15 vs H9N2-ZJ19, *p=0.0425 for PBS vs H9N2-ZJ19 in H9 protein testing, ****p<0.0001 for each pairwise comparation in H6 protein testing). N=10 in ≤0-5 yo group, 6-10 yo group, 11-20 yo group and 21-39 yo group, n=12 in 40-64 yo group, n=18 for ≥65 yo group from Guangzhou cohort. Total sample size; n=43 from CARES cohort, n=17 in the 60-69 yo group, n=18 in the 70-79 yo group and n=8 in 80-88 yo group. N=5 mice in per group except for the mice immunized with H1 stalk and H3 stalk which had 3 mice.

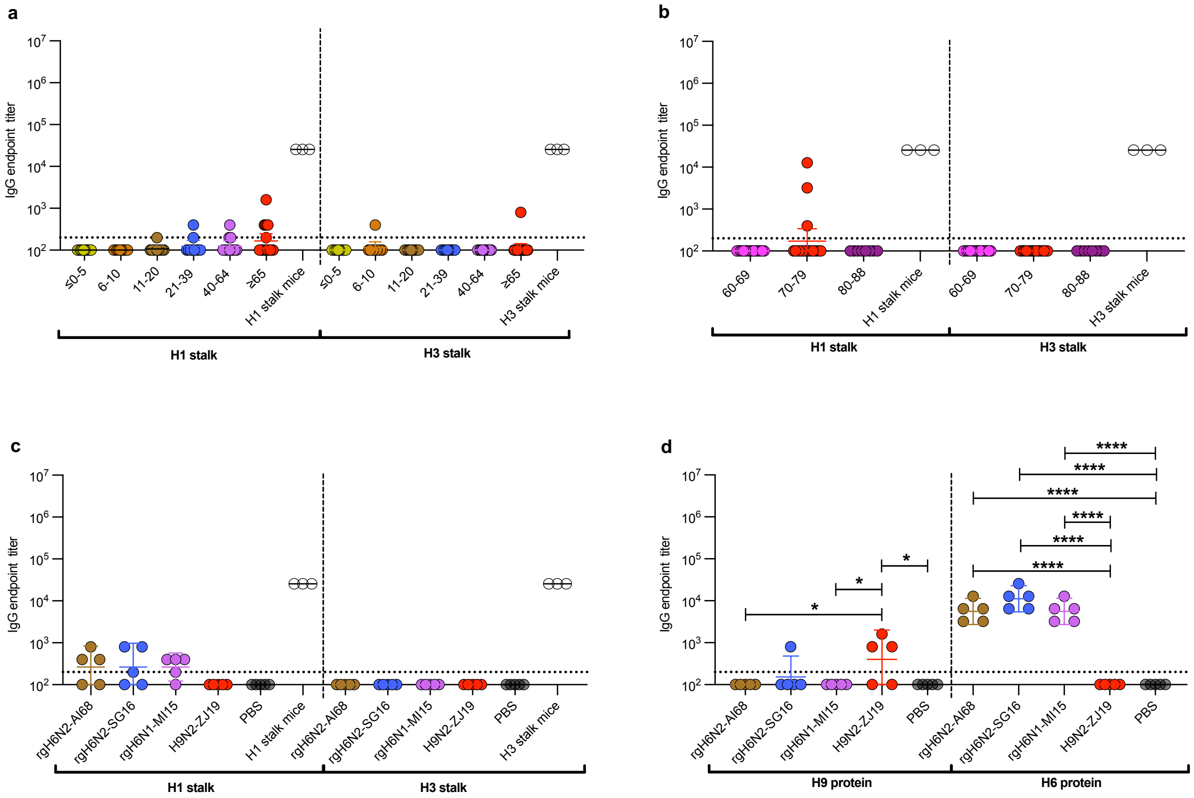

a
b
c
d
IgG endpoint titer
≤0-5
6-10
11-20
21-39
40-64
≥65
H1 stalk mice
H3 stalk mice
60-69
70-79
80-88
H1 stalk
H3 stalk
rgH6N2-AI68
rgH6N2-SG16
rgH6N1-MI15
H9N2-ZJ19
PBS
H9 protein
H6 protein
*
****

Supplementary Fig.6 | The neuraminidase-inhibition antibody profile against the other avian NA subtypes from CARES cohort. The neuraminidase-inhibition (NI) antibody profile against the N3 of A/Duck/Guangdong/1/1996 (H7N3), N5 of A/duck/Guangdong/wy11/2008 (H5N5), N7 of A/duck/Shanxi/3180/2010 (H10N7) and N9 of A/Anhui/1/2013 (H7N9), as determined for Fig.5, when stratified by year of birth (n=43).

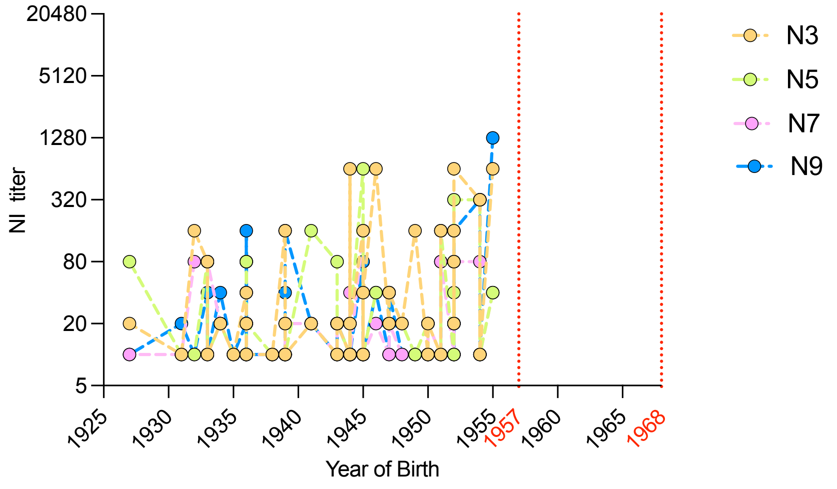

Supplementary Table 1 | Subtype A(H9N2) influenza viruses representative of each clade used in serology studies.

Strain	Abbreviation	Lineage
A/chicken/Hong Kong/G9/1997	HK97	BJ94-G9
A/guinea fowl/Hong Kong/WF10/1999	HK99	G1
A/chicken/Fujian/9290/2005	FU05	BJ94-G9
A/chicken/Hong Kong/YU250W/2011	HK11	G1
A/Pakistan/486/2015	PA15	G1
A/chicken/Egypt /S12568C/2016	EG16	G1
A/Suzhou/GIRD01/2019	SZ19	BJ94-F98
A/Oman/2747/2019	OM19	G1

Supplementary Table 2 | Age-stratified sampling scheme including viruses in circulation at the time of birth.

Age group (years of age)	n	Birth year range	Representative influenza A virus strains in circulation at time of birth^
≤5	20	2015-2020	A/Singapore/INFIMH160019/2016*<br>A/Kansas/14/2017<br>A/Switzerland/8060/2017
6-10	20	2010-2014	A/Perth/16/2009<br>Victoria/361/2011*<br>A/Texas/50/2014<br>A/Hong Kong/4801/2014*
11-20	20	2000-2009	A/Fujian/411/2002*<br>A/Wisconsin/67/2005<br>A/California/04/2007<br>A/Brisbane/10/2007*
21-39	20	1981-1999	A/Sichuan/2/1987*<br>A/Beijing/352/1989<br>A/Beijing/32/1992<br>A/Wuhan/359/1995*<br>A/Sydney/5/1997*<br>A/Moscow/10/99
40-64	20	1957-1980	A(H2N2), A/Aichi/2/1968 (Pandemic 1968)*<br>A/England/42/1972<br>A/Texas/1/1977<br>A/Bangkok/1/1979*<br>A/Beijing/39/1975<br>A/Victoria/3/1975*
≥65	20	1918-1956	A(H1N1) in circulation

^strains are of A(H3N2) subtype unless indicated otherwise
*antigenically-distinct subtype A(H3N2) strains selected for this study.

Supplementary Table 3 | Age-stratified hemagglutinin-inhibition antibody titers against representative subtype A(H9N2) and A(H3N2) influenza virus strains.

		Geometric mean titers (95% confidence intervals) measured in each age group					
**Subtype**	**Strain**	**≤5 years old**	**6-10 years old**	**11-20 years old**	**21-39 years old**	**40-64 years old**	**≥65 years old**
H9N2	HK97	5.2 (4.8-5.6)	5.4 (4.8-5.9)	5.0 (5.0-5.0)	5.2 (4.8-5.6)	5.0 (5.0-5.0)	5.0 (5.0-5.0)
	HK99	6.2 (5.1-7.4)	5.2 (4.8-5.6)	5.0 (5.0-5.0)	5.0 (5.0-5.0)	5.0 (5.0-5.0)	6.2 (5.1-7.4)
	FU05	5.4 (4.8-5.9)	5.4 (4.8-5.9)	5.0 (5.0-5.0)	5.0 (5.0-5.0)	5.2 (4.8-5.6)	5.2 (4.8-5.6)
	HK11	5.0 (5.0-5.0)	5.2 (4.8-5.6)	5.0 (5.0-5.0)	5.0 (5.0-5.0)	5.0 (5.0-5.0)	6.8 (5.3-8.7)
	PA15	5.7 (5.0-6.6)	5.9 (5.1-6.9)	5.0 (5.0-5.0)	5.4 (4.8-5.9)	5.4 (6.2-4.6)	5.0 (5.0-5.0)
	EG16	5.0 (5.0-5.0)	5.4 (4.8-5.9)	5.0 (5.0-5.0)	5.0 (5.0-5.0)	5.0 (5.0-5.0)	5.2 (4.8-5.6)
	SZ19	5.9 (4.8-7.3)	5.4 (4.8-5.9)	5.0 (5.0-5.0)	5.4 (6.2-4.6)	5.5 (4.7-6.5)	5.2 (4.8-5.6)
	OM19	6.6 (5.6-7.8)	5.5 (4.9-6.2)	5.2 (4.8-5.6)	5.2 (4.8-5.6)	5.5 (4.9-6.2)	5.7 (5.0-6.6)
H3N2	AI68	5.5 (4.9-6.2)	5.2 (4.8-5.6)	5.0 (5.0-5.0)	5.0 (5.0-5.0)	10.7 (6.5-17.7)	16.8 (10.3-27.5)
	VI75	6.8 (5.8-8.1)	5.4 (4.8-5.9)	5.0 (5.0-5.0)	5.2 (4.8-5.6)	9.0 (5.8-14.0)	10.7 (6.4-17.9)
	BK79	5.2 (4.8-5.6)	5.0 (5.0-5.0)	5.0 (5.0-5.0)	5.2 (4.8-5.6)	6.2 (5.1-7.4)	6.2 (5.0-7.6)
	SI87	5.2 (4.8-5.6)	5.0 (5.0-5.0)	5.5 (4.9-6.2)	8.7 (5.7-13.2)	11.1 (7.8-15.8)	10.7 (7.3-15.6)
	WU95	5.4 (4.8-5.9)	6.2 (5.3-7.2)	7.1 (4.7-10.6)	80.0 (41.1-155.7)	29.3 (18.9-45.5)	27.3 (17.0-44.0)
	SY97	5.0 (5.0-5.0)	5.2 (4.8-5.6)	32.5 (16.8-62.7)	105.6 (56.4-197.5)	26.4 (15.9-43.9)	28.3 (16.6-48.1)
	FU02	6.4 (4.7-8.6)	14.1 (7.7-26.0)	74.6 (36.0-154.6)	58.6 (32.8-104.)	13.2 (8.3-21.0)	18 (10.4-31.2)
	BR07	9.0 (8.0-10.1)	9.3 (7.4-11.8)	72.1 (35.8-145.2)	52.8 (31.4-88.8)	12.7 (8.2-19.7)	15.7 (9.7-25.5)
	VI11	21.4 (10.8-42.4)	37.3(17.6-79.1)	51.8 (31.4-52.7)	14.1 (8.9-22.5)	11.1 (7.6-16.3)	12.3 (8.5-17.8)
	HK14	20.7 (10.4-41.1)	25.5(12.4-52.6)	33.6 (19.7-57.5)	10.4 (6.6-16.3)	8.7 (6.2-12.3)	9.0 (6.5-12.4)
	SG16	18.7 (9.1-38.1)	29.3(13.5-63.7)	36.1(20.4-63.7)	11.9 (7.0-20.3)	9.0 (6.2-13.0)	10.4(7.1-15.0)

Supplementary Table 4 | Age-stratified neuraminidase-inhibition antibody titers against representative subtype A(H9N2) and A(H3N2) influenza virus strains.

		Geometric mean titers (95% confidence intervals) measured in each age group					
**Subtype**	**Strain**	**≤5 years old**	**6-10 years old**	**11-20 years old**	**21-39 years old**	**40-64 years old**	**≥65 years old**
H9N2	HK97	11.1 (9.9-12.5)	22.2 (15.8-31.1)	19.3 (13.5-27.6)	26.4 (17.2-40.4)	36.1 (23.3-55.8)	538.2 (302.7-956.7)
	HK99	18.0 (13.8-23.5)	65.0 (54.0-78.2)	44.4 (29.8-66.1)	74.6 (50.4-110.5)	91.9 (61.5-137.4)	502.1 (261.8-963.0)
	FU05	11.5 (10.1-13.1)	33.6 (25.0-45.2)	18.7 (13.6-25.5)	22.2 (16.1-30.6)	47.6 (28.8-78.7)	407.9 (226.4-734.7)
	HK11	14.1 (11.0-18.1)	49.2 (37.2-65.2)	30.3 (19.5-47.0)	45.9 (28.8-73.2)	80.0 (48.3-132.5)	485.0 (274.1-858.2)
	PA15	24.6 (19.0-31.9)	77.3 (65.5-91.2)	37.3 (23.9-58.3)	82.8 (52.1-131.8)	117.1 (67.5-203.4)	685.9 (393.4-1196.0)
	EG16	18.0 (14.5-22.4)	65.0 (54.0-78.2)	30.3 (19.5-47.0)	45.9 (28.8-73.2)	74.6 (45.1-123.5)	618.2 (384.0-995.3)
	SZ19	20.7 (15.8-27.1)	74.6 (59.1-94.2)	33.6 (21.3-53.1)	42.9 (27.5-66.9)	52.8 (32.8-84.9)	251.1 (147.9-426.2)
	OM19	19.3 (14.8-25.3)	72.1 (59.6-87.2)	38.6 (25.9-57.7)	51.0 (35.3-73.7)	67.3 (42.1-107.6)	437.1 (244.5-781.5)
H3N2	AI68	12.7(9.3-17.6)	20.7(15.8-27.1)	23.0(14.6-36.2)	74.6(43.2-128.8))	259.9(136.9-493.5)	787.9(422.4-1469.9)
	VI75	10.7(9.3-12.4)	26.4(19.8-35.1)	19.3(12.8-29.2)	85.7(40.8-180.3)	259.9(175.1-385.9)	171.5(95.5-307.8)
	BK79	12.7(10.5-15.4)	45.9(33.2-63.7)	30.3(17.3-53.1)	105.6(54.5-204.4)	367.6(239.4-564.4)	160.0(98.8-259.2)
	SI87	11.1(9.5-13.0)	28.3(20.1-39.8)	23.8(14.2-39.8)	88.8(43.0-183.0)	269.1(168.3-430.2)	109.3(66.4-180.0)
	WU95	18.0(11.4-28.6)	32.5(27.0-39.1)	80.0(43.3-147.8)	394.0(240.2-646.2)	177.5(131.2-240.3)	234.3(120.9-453.8)
	SY97	18.7(11.8-29.5)	27.3(22.5-33.2)	77.3(39.2-152.2)	380.5(227.5-636.4)	197(129.1-300.5)	251.1(125.6-501.7)
	FU02	30.3(18.6-49.3)	113.1(81.8-156.5)	519.8(332.2-813.5)	538.2(332.7-870.5)	269.1(190.2-380.7)	320.0(199.9-512.4)
	BR07	34.8(20.0-60.5)	88.8(51.8-152.2)	519.8(345.3-782.5)	211.1(119.3-373.6)	95.1(64.3-140.8)	139.3(85.4-227.2)
	VI11	33.6(20.1-56.3)	105.6(64.9-171.8)	485.0(294.7-798.2)	171.5(101.4-289.9)	67.3(44.2-102.3)	102.0(63.5-163.8)
	HK14	36.1(19.3-67.4)	165.6(108.1-253.9)	380.5(238.0-608.4)	177.5(98.5-319.8)	62.8(39.1-100.8)	72.1(44.4-117.2)
	SG16	52.8(29.0-96.1)	102.0(71.6-145.2)	67.3(42.6-106.3)	67.3(46.1-98.1)	33.6(27.3-41.4)	41.4(29.0-59.1)

Supplementary Table 5 | Correlation of N2 titers between AI68 with avian N2 in the ≥65-year-old and the 40-64-year-old age groups, determined using two-sided test by Pearson's correlation test. The correlation coefficient is indicated by r and error indicated by the 95% confidence interval. Statistically significant correlation is indicated by *, p<0.05; **, p<0.01; ***, p<0.001; and ****, p<0.0001.

**Age group**		**AI68 vs. HK97**	**AI68 vs. HK99**	**AI68 vs. FU05**	**AI68 vs. HK11**	**AI68 vs. PA15**	**AI68 vs. EG16**	**AI68 vs. SZ19**	**AI68 vs. OM19**
⩾65 years old	r	0.8463	0.7947	0.703	0.8965	0.8773	0.9273	0.8096	0.8646
	95% confidence interval	0.6454 to 0.9376	0.5432 to 0.9153	0.3781 to 0.8737	0.7525 to 0.9587	0.7107 to 0.9507	0.8222 to 0.9713	0.5720 to 0.9219	0.6836 to 0.9454
	P (two-tailed)	<0.0001 ****	<0.0001 ****	0.0005 ***	<0.0001 ****	<0.0001 ****	<0.0001 ****	<0.0001 ****	<0.0001 ****
40-64 years old	r	0.2068	0.4264	0.1504	0.5135	0.5632	0.4736	0.6693	0.71
	95% confidence interval	-0.2594 to 0.5949	-0.01987 to 0.7310	-0.3130 to 0.5559	0.09190 to 0.7790	0.1608 to 0.8051	0.03935 to 0.7574	0.3222 to 0.8578	0.3899 to 0.8770
	P (two-tailed)	0.3816	0.0608	0.5269	0.0206 *	0.0097 **	0.0349 *	0.0012 **	0.0005 ***

Supplementary Table 6 |Fold change of neuraminidase-inhibition antibody titers against priming viruses after challenge with ZJ19.

Immunization group	H3N2-AI68	rgH3N2-SG16	rgH1N1-MI15	rgH6N2-AI68	rgH6N2-SG16	rgH6N1-MI15	H9N2-ZJ19
Fold change	4	1	0.5	4	0.5	1	8
	8	1	1	8	1	1	4
	4	2	2	4	2	1	4
	4	1	0.25	8	2	0.5	2
	4	2		4	2	1	2

Supplementary Table 7 | Virus strains used to clone hemagglutinin (HA) and/or neuraminidase (NA) genes used in this study.

Gene	Strain	Abbreviation	Accession number
HA	A/Aichi/2/1968 (H3N2)	AI68	KF874500.2
	A/Victoria/3/1975 (H3N2)	VI75	CY121197.1
	A/Bangkok/1/1979 (H3N2)	BK79	CY114429.1
	A/Sichuan/2/1987 (H3N2)	SI87	CY121293.1
	A/Wuhan/359/1995 (H3N2)	WU95	KM821302.1
	A/Sydney/5/1997 (H3N2)	SY97	CY112885.1
	A/Fujian/411/2002 (H3N2)	FU02	KM821324.1
	A/Brisbane/10/2007 (H3N2)	BR07	MW298188.1
	A/Victoria/361/2011 (H3N2)	VI11	KM821347.1
	A/Hong Kong/4801/2014 (H3N2)	HK14	MW298183.1
	A/Singapore/INFIMH160019/2016 (H3N2)	SG16	MW298182.1
	A/chicken/Hong Kong/G9/1997 (H9N2)	HK97	KF188366.1
	A/guinea fowl/Hong Kong/WF10/1999 (H9N2)	HK99	KX859321.1
	A/chicken/Fujian/9290/2005 (H9N2)	FU05	CY023640.1
	A/chicken/Hong Kong/YU250W/2011 (H9N2)	HK11	KF259132.1
	A/Pakistan/486/2015 (H9N2)	PA15	MH930834.1
	A/chicken/Egypt/S12568C/2016 (H9N2)	EG16	KY558857.1
	A/Suzhou/GIRD01/2019 (H9N2)	SZ19	MT875137.1
	A/Oman/2747/2019 (H9N2)	OM19	EPI ISL 353983*
NA	A/Aichi/2/1968 (H3N2)	AI68	CY121119.1
	A/Victoria/3/1975 (H3N2)	VI75	CY121199.1
	A/Bangkok/1/1979 (H3N2)	BK79	CY114431.1
	A/Sichuan/2/1987 (H3N2)	SI87	CY112398.1
	A/Wuhan/359/1995 (H3N2)	WU95	CY112823.1
	A/Sydney/5/1997 (H3N2)	SY97	AJ291403.1

A/Fujian/411/2002 (H3N2)	FU02	CY112935.1
A/Brisbane/10/2007 (H3N2)	BR07	CY116578.1
A/Victoria/361/2011 (H3N2)	VI11	KJ942682.1
A/Hong Kong/4801/2014 (H3N2)	HK14	MW298250.1
A/Singapore/INFIMH160019/2016 (H3N2)	SG16	MW298249.1
A/chicken/Hong Kong/G9/1997 (H9N2)	HK97	AF156391.1
A/guinea fowl/Hong Kong/WF10/1999 (H9N2)	HK99	KX859316.1
A/chicken/Fujian/9290/2005 (H9N2)	FU05	CY023642.1
A/chicken/Hong Kong/YU250W/2011 (H9N2)	HK11	KF259534.1
A/Pakistan/486/2015 (H9N2)	PA15	MF280171.1
A/chicken/Egypt/S12568C/2016 (H9N2)	EG16	KY558854.1
A/Suzhou/GIRD01/2019 (H9N2)	SZ19	MT875139.1
A/Oman/2747/2019 (H9N2)	OM19	EPI ISL 353983*
A/chicken/Zhejiang/198/2019 (H9N2)	ZJ19	MT875147.1
A/duck/Guangdong/1/1996 (H7N3)	GD96	JQ988866.1
A/duck/Guangdong/wy11/2008 (H5N5)	GD08	CY091629.1
A/duck/Shanxi/3180/2010 (H10N7)	SX10	KU921408.1
A/Anhui/1/2013 (H7N9)	AH13	MW298223.1
A/Singapore/1/1957 (H2N2)	SG57	MW298226.1

*Sequence obtained from GISAID

Supplementary Table 8 | Seroconversion rates to the different NA subtypes among the 29 A(H3N2)-infected participants in CARES cohort.

NA Group	Group 1	Group 2						
Subtype	N5	Human N2		Avian N2		N3	N7	N9
Strain	GD08[b]	HK14[c]	AI68[d]	HK99[e]	PA15[f]	GD96[g]	SX10[h]	AH13[i]
Seroconversion[a] No. (%)	5(17.2)	22(75.9)	12(41.4)	8(27.6)	10(34.5)	7(24.1)	1(3.4)	2(6.9)

[a] Seroconversion was defined as four-fold increase in neuraminidase inhibition (NI) antibody titers in paired serum samples
[b] H6N5-A/duck/Guangdong/wy11/2008 (H5N5)
[c] H6N2_A/Hong Kong/4801/2014 (H3N2)
[d] H6N2_A/Aichi/2/1968 (H3N2)
[e] H6N2_A/Guineafowl/Hong Kong/WF10/99 (H9N2)
[f] H6N2_A/Pakistan/486/2015 (H9N2)
[g] H6N3_A/Duck/Guangdong/1/1996 (H7N3)
[h] H6N7_A/duck/Shanxi/3180/2010 (H10N7)
[i] H6N9_A/Anhui/1/2013 (H7N9)
