# Supplementary material for: A(H2N2) and A(H3N2) influenza pandemics elicited durable cross-reactive and protective antibodies against avian N2 neuraminidases
Source: Nat Commun. 2024 Jul 3;15:5593. doi: 10.1038/s41467-024-49884-9 (PMC11222539; doi:10.1038/s41467-024-49884-9)
Supplement: Supplementary file 3 — Reporting Summary [file 41467_2024_49884_MOESM3_ESM.pdf]

Corresponding author(s): Mark Zanin, Sook-San Wong

Last updated by author(s): May 31, 2024

# Reporting Summary

Nature Portfolio wishes to improve the reproducibility of the work that we publish. This form provides structure for consistency and transparency in reporting. For further information on Nature Portfolio policies, see our Editorial Policies and the Editorial Policy Checklist.

## Statistics

For all statistical analyses, confirm that the following items are present in the figure legend, table legend, main text, or Methods section.

| n/a | Confirmed | |
|---|---|---|
| ☐ | ☒ | The exact sample size (*n*) for each experimental group/condition, given as a discrete number and unit of measurement |
| ☐ | ☒ | A statement on whether measurements were taken from distinct samples or whether the same sample was measured repeatedly |
| ☐ | ☒ | The statistical test(s) used AND whether they are one- or two-sided<br>*Only common tests should be described solely by name; describe more complex techniques in the Methods section.* |
| ☐ | ☒ | A description of all covariates tested |
| ☐ | ☒ | A description of any assumptions or corrections, such as tests of normality and adjustment for multiple comparisons |
| ☐ | ☒ | A full description of the statistical parameters including central tendency (e.g. means) or other basic estimates (e.g. regression coefficient) AND variation (e.g. standard deviation) or associated estimates of uncertainty (e.g. confidence intervals) |
| ☐ | ☒ | For null hypothesis testing, the test statistic (e.g. *F*, *t*, *r*) with confidence intervals, effect sizes, degrees of freedom and *P* value noted<br>*Give P values as exact values whenever suitable.* |
| ☒ | ☐ | For Bayesian analysis, information on the choice of priors and Markov chain Monte Carlo settings |
| ☒ | ☐ | For hierarchical and complex designs, identification of the appropriate level for tests and full reporting of outcomes |
| ☒ | ☐ | Estimates of effect sizes (e.g. Cohen's *d*, Pearson's *r*), indicating how they were calculated |

*Our web collection on statistics for biologists contains articles on many of the points above.*

## Software and code

Policy information about availability of computer code

| | |
|---|---|
| Data collection | For phylogenetic analyses, Maximum likelihood phylogenetic trees were inferred using the maximum likelihood method with the GTR+G+I nucleotide substitution model in MEGA-X. |
| Data analysis | Statistical analyses were performed using GraphPad Prism (version 9), while correlations between different strains were performed by using corrplot package (version 0.92) (https://github.com/taiyun/corrplot) and Complex Heatmap was generated by using Complex Heatmap package (version 2.13.1)(http://bioconductor.org/packages/release/BiocViews.html#___Software ) using R software (version 4.2.2). |

For manuscripts utilizing custom algorithms or software that are central to the research but not yet described in published literature, software must be made available to editors and reviewers. We strongly encourage code deposition in a community repository (e.g. GitHub). See the Nature Portfolio guidelines for submitting code & software for further information.

## Data

Policy information about availability of data

All manuscripts must include a data availability statement. This statement should provide the following information, where applicable:

- Accession codes, unique identifiers, or web links for publicly available datasets
- A description of any restrictions on data availability
- For clinical datasets or third party data, please ensure that the statement adheres to our policy

The data generated in this study are provided in the Supplementary Information/Source Data file. Source data supporting the findings of this study are available on figshare at https://doi.org/10.6084/m9.figshare.25582482.

## Research involving human participants, their data, or biological material

Policy information about studies with human participants or human data. See also policy information about sex, gender (identity/presentation), and sexual orientation and race, ethnicity and racism.

| | |
|---|---|
| Reporting on sex and gender | Sex information was collected from participants in the three human cohorts for which serum samples were used. In each cohort, sex information was self-reported. For the Guangzhou Cohort and EPI-HK cohort, we pre-specified the sample collection criteria so that there was 1:1 ratio of males to females. Total participants included; 120 males and 120 females. In the CARES cohort, we used serum from individuals who were infected with influenza A virus, regardless of sex. Total 12 makes and 31 females were included. This information is provided as Table 1 in the manuscript. No sex or gender specific analyses were performed as it was not a main focus of our study. |
| Reporting on race, ethnicity, or other socially relevant groupings | Race, ethnicity or other socially relevant classifications were not reported in the present study. |
| Population characteristics | Our study was focused on the age-specific seroprevalence of influenza antibodies. Hence serum samples from Guangzhou Cohort and EPI-HK were collected from pre-defined age categories based on expected exposure history to influenza virus. |
| Recruitment | For the Guangzhou cohort, no active recruitment was performed as we used residual serum submitted to the Department of Laboratory Medicine. For EPI-HK, which was designed as a community-based longitudinal cohort study on individual and population immunity against respiratory virus infections in Hong Kong, individuals across all-ages were enrolled from the community starting in July 2020. For the analyses included here, we selected 120 individuals in each of the pre-specified age-groups. |
| Ethics oversight | This study received ethical approval from the Institutional Review Board of the First Affiliated Hospital of Guangzhou Medical University (Ref: ES-2023-K011-01). CARES received ethical approval from the Institutional Review Board of the University of Hong Kong (Ref: UW15 404) and the Ethics Committee of Jiangsu Provincial Center for Disease Prevention and Control (Ref: JSJK2015-B013-02). EPI-HK received ethical approval from Institutional Review Board of the University of Hong Kong (Ref: UW 19-720). |

Note that full information on the approval of the study protocol must also be provided in the manuscript.

# Field-specific reporting

Please select the one below that is the best fit for your research. If you are not sure, read the appropriate sections before making your selection.

☒ Life sciences ☐ Behavioural & social sciences ☐ Ecological, evolutionary & environmental sciences

For a reference copy of the document with all sections, see nature.com/documents/nr-reporting-summary-flat.pdf

# Life sciences study design

All studies must disclose on these points even when the disclosure is negative.

| | |
|---|---|
| Sample size | No sample size calculations were performed for the serosurvey cohort. Sample size were determined based on previous studies or published literature. |
| Data exclusions | Individuals with HAI-titer >40 to A(H9N2) were excluded from further analyses to ensure that the cross-reactivity observed were not due to undocumented past exposures to A(H9N2). |
| Replication | We validated our seroprevalence data in Figure 2 using samples from an independent cohort, based in Hong Kong. Data were reproducible in these two cohorts. |
| Randomization | Mice were randomly allocated into experimental group prior to the start of experiments. For serosurvey study using Guangzhou Healthy Cohort and EPI-HK samples, sera were collected based on pre-specified age and sex ratios. For the CARES study, samples were collected from |

| | |
|---|---|
| | PCR-positive participants, no randomization is relevant here. |
| Blinding | Blinding was not relevant in our study as this is largely an observational study with objective outcomes determined from laboratory assays. Further, it was not possible in our study as the laboratory assays were designed to test equal number of samples in each age groups per run to minimize batch effects. |

# Reporting for specific materials, systems and methods

We require information from authors about some types of materials, experimental systems and methods used in many studies. Here, indicate whether each material, system or method listed is relevant to your study. If you are not sure if a list item applies to your research, read the appropriate section before selecting a response.

## Materials & experimental systems

| n/a | Involved in the study |
|---|---|
| ☐ | ☒ Antibodies |
| ☐ | ☒ Eukaryotic cell lines |
| ☒ | ☐ Palaeontology and archaeology |
| ☐ | ☒ Animals and other organisms |
| ☐ | ☒ Clinical data |
| ☒ | ☐ Dual use research of concern |
| ☒ | ☐ Plants |

## Methods

| n/a | Involved in the study |
|---|---|
| ☒ | ☐ ChIP-seq |
| ☒ | ☐ Flow cytometry |
| ☒ | ☐ MRI-based neuroimaging |

## Antibodies

| | |
|---|---|
| Antibodies used | Anti-human (Bioss, bs-0297G-HRP) or anti-mouse IgG (Bioss, bs-0296G-HRP) secondary antibody. |
| Validation | This two antibodies are commercially purchased. |

## Eukaryotic cell lines

Policy information about cell lines and Sex and Gender in Research

| | |
|---|---|
| Cell line source(s) | 293T and Marin-Darby Canine Kidney cells |
| Authentication | The cell lines were not authenticated. |
| Mycoplasma contamination | The cell lines are mycoplasma-free, as tested by PCR. |
| Commonly misidentified lines (See ICLAC register) | Not applicable |

## Animals and other research organisms

Policy information about studies involving animals; ARRIVE guidelines recommended for reporting animal research, and Sex and Gender in Research

| | |
|---|---|
| Laboratory animals | We used 6-8 weeks of female Balb/c mice in this study as they develop more robust antibody IgG responses compared to male mice. Mice were purchased from Zhejiang Vital River Laboratory Animal Technology Co., Ltd and housed in the Experimental Animal Center of Guangzhou Medical University under in a ventilated isocage, in a room with a 12-h light/dark cycle. The temperature and humidity are maintained at 20-26[illegible]C with 30%-70% percent humidity. Food and water were provided ad libitum. |
| Wild animals | The study did not involve wild animals. |
| Reporting on sex | We used 6-8 weeks of female Balb/c mice in this study as they develop more robust antibody IgG responses compared to male mice. |
| Field-collected samples | The study did not involve samples collected from the field. |
| Ethics oversight | All mouse experiments were conducted in accordance with institutional animal care guidelines and were approved by the Animal Care Committee of Guangzhou Medical University. |

Note that full information on the approval of the study protocol must also be provided in the manuscript.

## Clinical data

Policy information about clinical studies

All manuscripts should comply with the ICMJE guidelines for publication of clinical research and a completed CONSORT checklist must be included with all submissions.

| | |
|---|---|
| Clinical trial registration | Not applicable |
| Study protocol | Not applicable |
| Data collection | Not applicable |
| Outcomes | Not applicable |

## Plants

| | |
|---|---|
| Seed stocks | Not relevant to our study |
| Novel plant genotypes | Not relevant to our study |
| Authentication | Not relevant to our study |
