# Supplementary material for: A(H2N2) and A(H3N2) influenza pandemics elicited durable cross-reactive and protective antibodies against avian N2 neuraminidases
Source: Nat Commun. 2024 Jul 3;15:5593. doi: 10.1038/s41467-024-49884-9 (PMC11222539; doi:10.1038/s41467-024-49884-9)
Supplement: Supplementary file 2 — Peer Review File [file 41467_2024_49884_MOESM2_ESM.pdf]

nature portfolio

Peer Review File

A(H2N2) and A(H3N2) influenza pandemics elicited durable cross-reactive and protective antibodies against avian A(H9N2) neuraminidases

Open Access This file is licensed under a Creative Commons Attribution 4.0 International License, which permits use, sharing, adaptation, distribution and reproduction in any medium or format, as long as you give appropriate credit to the original author(s) and the source, provide a link to the Creative Commons license, and indicate if changes were made. In the cases where the authors are anonymous, such as is the case for the reports of anonymous peer reviewers, author attribution should be to 'Anonymous Referee' followed by a clear attribution to the source work. The images or other third party material in this file are included in the article's Creative Commons license, unless indicated otherwise in a credit line to the material. If material is not included in the article's Creative Commons license and your intended use is not permitted by statutory regulation or exceeds the permitted use, you will need to obtain permission directly from the copyright holder. To view a copy of this license, visit http://creativecommons.org/licenses/by/4.0/.

Reviewers' Comments:

Reviewer #1:
Remarks to the Author:
In their manuscript Liang and colleagues investigated N2 NA crossreactivity between human N2 and avian N2. The work is very interesting and timely right now since there is a lot of attention on NA immunity. However, there are several points that need the authors' attention.

Major points

1) N2 NA from H2N2 was completely left out of the analysis but needs to be included.

2) For the H9N2 passive transfer challenge experiments, the authors need to assess anti-stalk and anti-H9 binding antibody titers for the different age groups. This is also true for the mouse immunization-challenge experiments.

3) Cross-NI activity found against different NA subtypes (e.g. line 242) could be cause by stalk reactive/crossreactive antibodies to the different HAs. This needs to be measured for all cases.

4) In general, the authors do not do a good job to indicate where wild type viruses were used, where H6NX viruses were used etc. This should be indicated very clearly in all figures. It allows the reader to understand where which type of HA antibody could have interfered with the assay.

Minor points

1) Line 64: Should be 'influenza virus subtypes'.

2) Line 78, 85: Should be 'influenza virus'. In general, whenever the virus is meant and not strictly the disease, it should be 'influenza virus'.

3) Line 106: 'admitted to the hospital'

4) Line 161: Since N2 from H2N2 was not included this is not really 'early'.

5) Line 296: How does this compare with other reports where binding antibody was assessed in infected humans? (e.g. https://pubmed.ncbi.nlm.nih.gov/28192418/)

6) Line 284: 'had a higher'

7) Line 308: 'novel HA subtypes'

8) Line 322 to 327: There is absolutely no indication that there is crossreactivity between two different NA subtypes just because both are 'avian'. Please remove this or back it up with appropriate references.

9) Line 340: 'was', not 'were'

10) Line 362: Define 'PFU'

11) Line 391: 'genes of interest', not 'genes of interests'

12) Line 418: Please describe in detail how the virus mount was determined and standardized.

13) Figure 1A: Please provide a scale bar.

14) Figure 1B: This figure is confusing and hard to understand.

15) Figure 4: Is there reason why these experiments were not performed with recombinant NA, which would be much cleaner?

16) Figure 6A: This figure is really hard to read/understand.

Reviewer #2:
Remarks to the Author:
Major points
1. This study analyzed the age-stratified seroprevalence of cross-reactive N2 antibodies. It is important to clarify how these age groups were determined (Supplementary Tabe 1) and whether the number of participants in each group was enough.
2. Lines 231-235: The authors mentioned the PCR detection of H3N2 for the 40 participants from Jiangsu. However, due to the high prevalence of H9N2 in Jiangsu and also the high sero-prevalence among humans, it is essential to provide more background information of these participants and to exclude that these participants had prior exposure to H9N2 or other HxN2.
3. The study involved several AIVs of both avian and human origins, as well as recombinant AIVs of different subtypes. However, the author did not clarify them clearly enough in the main text and also in the figures. The improper use of abbreviations of the test strains made the text extremely hard to follow.
4. Lines 50-51: "Seasonal A(H3N2) infection could recall these AIV N2 antibodies, showing distinct human- or avian NA-type reactivity, in 19-26% of individuals."
I did not find these estimates from the main text. 19-26% of whom? All age groups? Please clarify.
5. Lines 246-248: 'N3 and N9 titers were also highest among 60-69yo, with titers significantly higher compared to younger adults.' Please discuss this in the Discussion section.

Minor points
Lines 47-48, please provide a reference for this statement.
Line 64, a reference is needed.
Line 113, Not sure how these 118 representative N2 sequences were selected. Please clarify.
Fig. 1a. Where is BJ/94? Please highlight this strain. Why strains of G1 fell within two lineages?
Fig. 1. Does panel a and b involve the same genome sequences?
Line 70, "A(H5N1) AIV cases are predominant among individuals <15 years old" Could this weird phenomena be explained by the lack of prior HxN1 AIV exposure?
Line 90: please append 'human-infecting' before 'A(H5N1) and A(H7N9)'
Line 176. Statistical analysis is needed for fig. 2f.

Reviewer #3:
Remarks to the Author:
Imprinting is known to exert profound population-level effects on the outcome of subsequent influenza virus infections (and vaccinations). Yet, the vast majority of data in the field has focused on imprinting by HA (at the exclusion of NA). NA-specific antibodies are known to confer protection from influenza virus infection, and NA is more immunogenic than HA on a molecule-per-molecule basis. The manuscript by Liang et al explores NA-specific imprinting conferred by the 1957 H2N2 pandemic and the 1968 H3N2 pandemic, and the impact of this imprinting on immunity to contemporary avian H9N2 viruses.

There is no doubt that this is a very important and potentially impactful topic. The data shown in Fig.2

convincingly show age-specific trends in NA cross-reactivity consistent with what has been previously described in the context of HA. However, the subsequent experiments performed to evaluate whether these NA-specific antibodies are truly cross-protective, and the extent of their recall, suffer from limitations that confound interpretation and limit the strength of conclusions that can be drawn. Specific remarks for the authors' consideration can be found below:

Major Concerns:

1. It is extremely difficult to follow/interpret the data shown in Fig. 3a-d due to a lack of clarity in both the figure legend and corresponding results section. These should be re-written to facilitate easier interpretation. Perhaps inclusion of experimental schema would be helpful.

2. In Fig. 3f, the authors rely on the conventional 4-fold change in titers as an indicator of seroconversion to support their argument regarding cross-reactivity of AI68 and ZJ19 N2. However, the absolute difference relative to SG16, or even MI15 is only slightly greater than 2-fold, which is hardly convincing.

3. Much of the data presented by the authors is potentially confounded by antibodies that may recognize HA. The authors perform HAI assays in several figures, however, antibodies that bind to the HA stalk are typically HAI-negative. Thus, it would be important to measure cross-reactive antibodies by either ELISA (to measure total cross-reactive binding), or a functional assay (for example, one that measures Fc-dependent effector functions) to evaluate their possible contribution. This concern is amplified by the fact that H6 and H9 are both group 1 HAs with highly conserved stalk domains. This confounder could be eliminated completely where the authors to immunize animals with purified, recombinant NA in several experiments.

4. In Fig.4, the authors claim that mice that received sera from rgH6N2-AI68 lost less weight than those that received sera from rgH6N2-SG16 however, this is not clear from the data shown in Fig.4a where there seems to be near identical weight loss and overlapping error bars. Indeed, the figure legend seems to indicate that statistical differences shown are relative to the PBS control group. In Fig. 4b the is evidently a difference between the two groups in terms of survival, but these could very well be stochastic given the similarity in the weight loss curves (since all animals appear to lose 20+% of initial body weight. Were 20% weight loss applied as the threshold for endpoint (which is the standard applied by many animal ethics committees), it does not seem that there would be any difference at all in survival.

Minor comments:

In several cases, the figure legends do not provide sufficient details to independently interpret the data shown in the corresponding figures. They could be improved by addition of more detail.

Point-by-point response

In particular, the revised manuscript will need to rule out a role of antibodies recognizing HA stalk, provide more detailed information on human participants, and comprehensively address all other concerns raised by our reviewers.

Response: We thank all the reviewers for their insightful comments to strengthen our manuscript. We have addressed these comments as detailed below.

REVIEWER COMMENTS

Reviewer #1 (Remarks to the Author):

In their manuscript Liang and colleagues investigated N2 NA cross reactivity between human N2 and avian N2. The work is very interesting and timely right now since there is a lot of attention on NA immunity. However, there are several points that need the authors' attention.

Major points

1) N2 NA from H2N2 was completely left out of the analysis but needs to be included.

Response: We thank the reviewer for this important comment. We did not include the N2 from H2N2 in our testing while in Guangzhou due to biosafety requirements. However, we have since obtained approval from our present institution's Biosafety Office. Due to the lack of sufficient serum samples remaining from the Guangzhou cohort and restrictions on exporting biological samples from Mainland China, we performed this study using serum samples collected from a community cohort in Hong Kong, EPI-HK. This also serves an additional cohort to validate our previous finding using the Guangzhou and CARES cohort. Information about EPI-HK have also been included into Table 1 in the revision. The results obtained were consistent with the Guangzhou and CARES cohorts samples and confirmed that the high avian N2 cross-reactivity observed in those born prior to 1957 tracked well with the N2 from A/Singapore/1/1957 (H2N2). We have incorporated these data as Figure 6d-i in the revision and as follows;

Page 15, Line 319: To evaluate whether the high avian N2 cross-reactivity was related to the 1957 pandemic exposure, we tested a subset of age-stratified serum samples collected from a large community-based cohort study on respiratory virus infections in Hong Kong, "Evaluating Population Immunity in Hong Kong" (EPI-HK) 25. After excluding 7 individuals with positive H9 HI titers against HK11 and SZ19 from 112 participants), we tested the remaining 105 samples for antibodies against N2 from A/Singapore/02/1957(H2N2) (SG57), AI68 and the two-representative avian N2, HK99 and PA15. Results from this cohort showed that N2 titers to SG57 were highest in those born before 1957 and gradually declined thereafter (Fig.6d). In contrast, N2 titers to AI68 were not different between those born before 1957 and those born between 1957 and 1968 (Fig.6e). This birth-year response pattern was consistent to that of HK99 and PA15 although the SG57 titers were

generally lower compared to the two avian strains (Fig.6f-h). The N2 titers were well-correlated amongst the tested strains but a stronger correlation was noted between the avian N2s with SG57 than AI68 (Fig.6i). Given that the AI68 N2 still shares antigenic similarity with the 1957 and avian N2, it is likely that exposure to the 1968 N2 elicited antibodies that can cross-react with avian N2 in naïve population while boosting the pre-existing N2 antibodies, resulting in higher avian N2 titers in the pre-1957 cohort.

2) For the H9N2 passive transfer challenge experiments, the authors need to assess anti-stalk and anti-H9 binding antibody titers for the different age groups. This is also true for the mouse immunization-challenge experiments.

Response: We thank the reviewer for this important comment. As suggested, we have tested for Group 1 and 2 HA-stalk as well as H9 antibodies in remaining serum samples from the Guangzhou and CARES cohort using ELISA. We used HA-stalk constructs that were previously described by our colleague and present collaborator (see references [1-3]). For the Guangzhou cohort, we found that, although there was a trend of increasing anti-H1 stalk antibodies, titers were not statistically significant across all age groups, and low titers were detected against Group 2-stalk (Supplemental Fig.3a).

In terms of anti-H9 antibodies, we measured IgG antibodies against a recombinant H9 protein from A/Hong Kong/1073/1999 (H9N2). H9-binding antibodies were detected in some individuals above the ages of 21. However, as a group, there were no statistically significant difference in their average titers except for those aged >65 yo when compared to 11-20 yo and <5 yo. Despite the trend of higher H9-antibody titers in 21-39 yo, 40-64 yo and >65 yo, no significant protection was observed following passive transfer of sera from 21-39 yo, suggesting to us that H9-binding antibodies were not strong determinants of the antibody cross-reactivity and protection observed in the mouse experiments.

As per reviewer's comments, we also tested the sera from the mouse immunization experiments for HA-stalk, H9 and H6 antibodies (Supplemental Figure 5). As expected, since H6 belongs to Group 1, mice immunized with the rgH6Nx constructs elicited more Group 1 HA stalk antibodies and no Group 2 HA stalk antibodies were detected. No antibody cross-reactivity was observed between H6 and H9 and no significant differences in anti-HA stalk antibodies and anti-H9 binding were detected across the different groups of immunized mice.

These changes have been incorporated into the manuscript as an additional result section and as Supplemental Figure 5:

Page 14, Line 287:

Role of HA-stalk and HA-binding antibodies

HA-stalk antibodies have been reported to interfere with NI assays through steric hindrance[21]. To exclude the potential interference by HA-stalk antibodies in our data, we measured the level of antibodies specific for Group 1 and 2 HA-stalk regions using previously described constructs, pH1HA10-Foldon (designed from A/California/04/2009 (H1N1)) and HK68-H3-SI (designed from A/Hong Kong/1/1968 (H3N2))[22-24] in the human and mice serum samples used in our study. Minimal antibodies were detected against both stalk proteins across all age groups in both the Guangzhou and CARES cohorts with no significant difference detected across any age groups (Supplemental Fig.5a, b). We also tested the sera from the mouse immunization experiments for HA-stalk, H9 and H6 antibodies. As expected, since H6 belongs to Group 1, mice immunized with the rgH6Nx constructs elicited more Group 1 HA stalk antibodies compared to Group 2, although the titers were still relatively low. No significant difference of anti-HA stalk antibodies and anti-H9 binding were detected across the different groups of immunized mice (Supplemental Fig.5 c, d). No cross-reactivity were observed between H6 and H9 (Supplemental Fig.5d). We conclude that HA-stalk and H9-binding antibodies are not major determinants of the cross-reactivity observed.

3) Cross-NI activity found against different NA subtypes (e.g. line 242) could be cause by stalk reactive/cross reactive antibodies to the different HAs. This needs to be measured for all cases.

Response: We have now included data for HA-stalk antibodies and H9-binding antibodies for human sera that were used in the study. We reasoned that any cross-reactive antibodies recognizing H9 will be detected using a H9-binding ELISA. As described above, although some Group 1 stalk antibodies were detected in the adult sera, these patterns of response were not consistent with the NA-cross-reactivity and mice protection data. Older adult from the CARES cohort also showed low titers of anti-stalk antibodies (Supplemental Fig.1 and 5). The revised changes are included as an additional results section as described in the response to comment 2 (Page 13, Line 285 in the revised manuscript).

Additionally, to exclude the potential interference by H9-binding antibodies on the cross-reactive NI activity, we re-analyzed our data using only individuals who were H9-seronegative by HAI and ELISA. Re-analyses of the Guangzhou Cohort data is included as Supplemental Figure 2 and for the CARES cohort data as Supplemental Figure 4 and Supplemental Table 8. In both re-analyses, our original pattern of cross-reactivity and its correlations still stand. This point is incorporated into our revised manuscript as follows:

For data describing Guangzhou Cohort, Page 9, Line 178:

To further exclude the role of HA-binding antibodies in our observation, whether from past A(H9N2) exposure or any cross-reactive antibodies that may target H9, we measured their H9-binding antibodies against recombinant H9 protein A/Hong Kong/1073/99 (H9N2) by ELISA. Some individuals above the ages of 21 showed H9-binding positivity (Supplemental Fig.1a). However, except for those aged ≥ 65yo compared to the 11-20yo and the ≤ 5yo, there were no statistically

significant difference amongst other age groups. Because pooled sera from the 21-39 yo did not protect the mice from the ZJ19 challenge (Fig 2f, g), this suggests that H9-binding antibodies were not a major determinant in the protective effect observed. Further, even after excluding those participants with anti-H9 binding positivity, all our observed pattern of cross-reactivity in the Guangzhou cohort still holds (Supplemental Fig.2), suggesting that this avian N2 cross-reactivity is not due to previous exposures to H9N2.

For data describing the CARES cohort, Page 14, Line 282:
Presence of cross-reactive H9 antibodies at baseline, as determined by either ELISA and HAI, did not seem to affect these NA responses as these differences and correlations still holds even when we restricted the analyses to the 29 H9-seronegative (by both assays) individuals (Supplemental Fig.4, Supplemental Table 8).

4) In general, the authors do not do a good job to indicate where wild type viruses were used, where H6NX viruses were used etc. This should be indicated very clearly in all figures. It allows the reader to understand where which type of HA antibody could have interfered with the assay.

Response: We thank the reviewer for identifying this issue. For the HI-assays, reverse-genetics (rg) (6+2) viruses were generated, except for A/Aichi/2/1968 (H3N2), A/guinea fowl/Hong Kong/WF10/1999 (H9N2) (HK99) and A/chicken/Zhejiang/198/2019 (H9N2), for which we had wild-type viruses. The rgHxNx viruses were generated with HA and NA of the target strain, with the 6-internal genes of A/Puerto Rico/8/1934 (H1N1)(PR8). For the NI-assay, rgH6Nx (7+1) viruses were generated for ELLA. The rgH6Nx viruses were generated with the target NA and the H6 from A/teal/Hong Kong/W312/1997 (H6N1) (7+1 configuration). This was included in the Method section, in the "Virus generation, propagation and titration" subsection (Page 23, Line 485). We have also now added this information in the figure legends where appropriate.

We also realized that the original Figure 3 was poorly written in the original manuscript, as was pointed out by the other reviewers. We have revised this section to improve clarity by separating the experiments described in Figure 3 into two parts. The first part describes the antigenic relationship between early human and avian N2 (page 10, Line 190, Figure 3) and the second part demonstrates the protective capacity of AI68 N2 antibodies against A(H9N2) challenge (page 11, Line 213, Figure 4). The first part uses wild-type or wild-type like viruses to study cross-reactivity. In the second part, we wanted to demonstrate in system without interference of HA immunity that the observed cross-reactivity can be protective. For this second part, we generated isogenic 7+1 viruses and only varying the NA segment. In this experiment, ZJ19 was the only wild-type virus used. Further, we included new experimental schema for each experiment (Figure 3a and Figure 4a(i) and (ii)), clearly indicating what viruses were used in the Figures and Figure legends. The revisions are as follows:

Page 10-11, Line 190-231:
Antigenic relationship between early human and avian N2.

To determine if exposure to early human N2s was a determinant of cross-reactivity, we performed a prime-challenge experiment in mice (Fig.3a). Mice was primed with A/Aichi/2/1968(H3N2) (AI68) wild type virus, and reverse-genetics derived viruses bearing the HA and NA of A/Singapore/INFIMH160019/2016 H3N2) (SG16) or A/Michigan/45/2015 (H1N1) (MI15). AI68 chosen to represent the early H3N2 virus while SG16 chosen to represent a contemporary H3N2 virus. MI15 was used as heterologous NA subtype control. Although the priming doses were predetermined to elicit comparable N2 titers between AI68 and SG16 (data not shown), in the eventual experiment, the two priming doses elicited higher homologous HI and NI titers in the AI68-primed mice compared to SG16 (Fig.3b). The control ZJ19 and rgH1N1 viruses had higher HI but lower NI titers compared to AI68.

We assessed the baseline cross-reactivity in the pre-challenge immune sera to the NA of ZJ19. Apart from the positive control, AI68 had the highest titers to ZJ19 and was significantly higher compared to SG16 and MI15 (Fig.3c). Post challenge, AI68- and ZJ19-primed groups showed smaller increases in ZJ19 N2 titers compared to SG16-, MI15- and PBS-primed groups (Fig.3c and d), likely due to the higher levels of baseline antibody. However, only mice in the AI68 or ZJ19 group showed significant induction of priming N2 antibodies and at least four-fold increases in NI-titers after challenge (Fig.3e and f, Supplemental Table 6). All animals were protected, with no significant differences in weight loss amongst groups (Supplemental Fig.3a, b, c), except for those in the PBS group. These data indicate that AI68 and ZJ19 shared cross-reactive epitopes and were antigenically closer compared to SG16.

AI68 immune sera were protective against ZJ19 challenge in mice.
To clearly demonstrate the role of NA and to reduce potential interference from the different HAs, we generated a complementary set of isogenic, reverse-genetics (rg)H6Nx viruses bearing the NA from AI68, SG16, or MI15 in the second prime-challenge experiment (Fig.4a(i)). After two priming doses, the HI titers were comparable amongst the rgH6Nx viruses but were significantly lower than the H9N2 control (Fig.4b) whereas the NI titers were comparable for N2 but lower than the rgH6N1 control. As in Fig. 3f, challenge with ZJ19 only boosted antibodies to AI68 and its homologous antigen (Fig. 4c and d). Except for mice in the PBS group, all animals survived the challenge (Supplemental Fig.3d, e, f).

To demonstrate that the antibodies elicited by the AI68 N2 were protective against ZJ19 challenge, we immunized another set of mice and conducted passive transfer of the immune sera into naïve mice prior to challenge (Fig.4a(ii)). Mice that received SG16 and MI15 immune sera lost significantly more weight than mice receiving AI68 N2-primed sera at one to seven days post-inoculation (Fig.4e). After day eight, surviving mice recovered their weight and were mostly indistinguishable from mice immunized with ZJ19. Despite some early weight loss, 70% of mice that received sera from AI68-primed mice survived compared to 0- 25% survival rate in the other groups (Fig.4f). These data indicate that N2 antibodies generated by AI68 priming were protective against lethal A(H9N2) AIV infection compared to contemporary human N2.

Reviewer #2 (Remarks to the Author):

Major points

1. This study analyzed the age-stratified seroprevalence of cross-reactive N2 antibodies. It is important to clarify how these age groups were determined (Supplementary Tabe 1) and whether the number of participants in each group was enough.

Response: We thank the reviewer for this comment. The age groups were determined based on the expected exposure to the different influenza viruses in circulation during early life, or immune imprinting, as clarified in Materials and Methods-Serology). This age-groups were designed based on previous findings that predicted probability of exposure[4]; i.e 0-5 yo: no or at most 1 exposure, 6-10 yo: at least 1 exposure, 11-20 yo: likely more than 1 exposure, 21-39 yo: multiple exposures. For the 40-64 yo and the >65 yo, we further considered the potential imprint profile due to pandemic exposure at time of birth. We determined the number of samples based on the findings of our previous studies where we were able to detect age-specific differences in antibody responses to HA and NA in cohorts of less than 10 participants per age group[5] and broad differences in the cellular immune responses in cohorts of 25 to 27 individuals per test groups[6]. The relevant details have been included in the Methods-Serology section as below:

Page 22, Line 461-468: In the Guangzhou cohort, twenty serum samples per age group were collected based on previous findings that predicted probability of exposure[2]; i.e 0-5 yo: no or at most 1 exposure, 6-10 yo: at least 1 exposure, 11-20 yo: likely more than 1 exposure, 21-39 yo: multiple exposures. For the 40-64 yo and the >65 yo, we further considered the potential imprint profile due to early life exposure based on the viruses in circulation at time of birth (Supplemental Table 2). In the EPI-HK cohort, we modified the age groups to 0-10, 11-20, 21-39, 40-64, 65-74 and >75 years old to prioritize the older adults. Both cohorts were pre-specified to include equal ratios of males to females.

2. Lines 231-235: The authors mentioned the PCR detection of H3N2 for the 40 participants from Jiangsu. However, due to the high prevalence of H9N2 in Jiangsu and also the high seroprevalence among humans, it is essential to provide more background information of these participants and to exclude that these participants had prior exposure to H9N2 or other HxN2.

Response: Participants were recruited from the community in Yancheng and Suzhou, Jiangsu province. We have provided additional information on the vaccination history and potential poultry exposure of the 40 participants that were PCR-positive for H3N2 in CARES as Table 1. In addition, we have also tested the baseline serum samples from these participants for H9 antibodies by hemagglutinin-inhibition assay (HAI) and ELISA. Five and nine individuals showed positive titers against any of the three A(H9N2) strains by HAI (11.6%) or ELISA (20.9%), respectively. No individuals had titers against any of the three A(H9N2) strains by both HAI and ELISA (Supplemental Fig.1b and 1c). In order to exclude the role of potential exposures to A(H9N2), we provided a re-analyses of Fig 5 and Table 2 that excluded participants with anti-H9 binding activity as

Supplemental Figure 4 and Supplemental Table 8. In this re-analyses of the 29 H9-negative participants, the cross reactive avian NA antibodies were still present in older adults and the original correlations were maintained. As there were no epidemiological reports of other avian HxN2 outbreaks, we think that it is unlikely that these individuals could have been exposed to such viruses, at least in recent years. However, based on our findings, we could not exclude the possibility that an avian N2 predecessor may have circulated in China prior to the A(H2N2) emergence in 1957. This was discussed in Line 374 of the Discussion section. The changes were incorporated in the revision as:

Page 12, Line 241:
These adults were infected with A(H3N2) viruses belonging to clade 3c.2a. This group of selected participants has no history of influenza vaccination and 37.2% of them reported visits to live poultry market within the last 12 months. At baseline, 5 of the 43 individuals (11.6%) have HI antibodies > 40 to at least one of three A(H9N2) strains and 9 of them (20.9%) have detectable H9 protein binding antibodies (Supplemental Fig.1b, c), although interestingly none were positive by both assay. None of the participants have HI antibodies to A(H7N9).

For re-analyses of H9-seronegative participants, Page 14, Line 282:
"Presence of cross-reactive H9 antibodies at baseline, as determined by either ELISA and HAI, did not seem to affect these NA responses as these differences and correlations still holds even when we restricted the analyses to the 29 H9-seronegative (by both assays) individuals (Supplemental Fig.4)."

3. The study involved several AIVs of both avian and human origins, as well as recombinant AIVs of different subtypes. However, the author did not clarify them clearly enough in the main text and also in the figures. The improper use of abbreviations of the test strains made the text extremely hard to follow.

Response: We thank the reviewer for bringing this to our attention. To clarify, for the HI-assays, reverse-genetics (rg) (6+2) viruses were generated, except for A/Aichi/2/1968 (H3N2), A/guinea fowl/Hong Kong/WF10/1999 (H9N2) (HK99) and A/chicken/Zhejiang/198/2019 (H9N2), for which we had the wild-type viruses. The rgHxNx viruses were generated with HA and NA of the target strain, with the 6-internal genes of A/Puerto Rico/8/1934 (H1N1)(PR8). For the NI-assay, rgH6Nx (7+1) viruses were generated for ELLA. The rgH6Nx viruses were generated with the target NA and the H6 from A/teal/Hong Kong/W312/1997 (H6N1) (7+1 configuration). This was included in the Method section, in the "Virus generation, propagation and titration" subsection (Page 23, Line 485). We have also now added this information in the figure legends where appropriate.

We also realized that the original Figure 3 was poorly written in the original manuscript, as was pointed out by the other reviewers. We have revised this section to improve clarity by separating the experiments described in Figure 3 into two parts. The first part describes the antigenic relationship between early human and avian N2 (page 10, Line 190, Figure 3) and the second part demonstrates the protective capacity of AI68 N2 antibodies against A(H9N2) challenge (page 11, Line 213, Figure

4). The first part uses wild-type or wild-type like viruses to study cross-reactivity. In the second part, we wanted to demonstrate in system without interference of HA immunity that the observed cross-reactivity can be protective. For this second part, we generated isogenic 7+1 viruses and only varying the NA segment. In this experiment, ZJ19 was the only wild-type virus used. Further, we included new experimental schema for each experiment (Figure 3a and Figure 4a(i) and (ii)), clearly indicating what viruses were used in the Figures and Figure legends.

The revisions are as follows:
Page 10-11, Line 190-231:
"Antigenic relationship between early human and avian N2.
To determine if exposure to early human N2s was a determinant of cross-reactivity, we performed a prime-challenge experiment in mice (Fig.3a). Mice was primed with A/Aichi/2/1968(H3N2) (AI68) wild type virus, and reverse-genetics derived viruses bearing the HA and NA of A/Singapore/INFIMH160019/2016 H3N2) (SG16) or A/Michigan/45/2015 (H1N1) (MI15). AI68 chosen to represent the early H3N2 virus while SG16 chosen to represent a contemporary H3N2 virus. MI15 was used as heterologous NA subtype control. Although the priming doses were predetermined to elicit comparable N2 titers between AI68 and SG16 (data not shown), in the eventual experiment, the two priming doses elicited higher homologous HI and NI titers in the AI68-primed mice compared to SG16 (Fig.3b). The control ZJ19 and rgH1N1 viruses had higher HI but lower NI titers compared to AI68.

We assessed the baseline cross-reactivity in the pre-challenge immune sera to the NA of ZJ19. Apart from the positive control, AI68 had the highest titers to ZJ19 and was significantly higher compared to SG16 and MI15 (Fig.3c). Post challenge, AI68- and ZJ19-primed groups showed smaller increases in ZJ19 N2 titers compared to SG16-, MI15- and PBS-primed groups (Fig.3c and d), likely due to the higher levels of baseline antibody. However, only mice in the AI68 or ZJ19 group showed significant induction of priming N2 antibodies and at least four-fold increases in NI-titers after challenge (Fig.3e and f, Supplemental Table 6). All animals were protected, with no significant differences in weight loss amongst groups (Supplemental Fig.3a, b, c), except for those in the PBS group. These data indicate that AI68 and ZJ19 shared cross-reactive epitopes and were antigenically closer compared to SG16.

AI68 immune sera were protective against ZJ19 challenge in mice.
To clearly demonstrate the role of NA and to reduce potential interference from the different HAs, we generated a complementary set of isogenic, reverse-genetics (rg)H6Nx viruses bearing the NA from AI68, SG16, or MI15 in the second prime-challenge experiment (Fig.4a(i)). After two priming doses, the HI titers were comparable amongst the rgH6Nx viruses but were significantly lower than the H9N2 control (Fig.4b) whereas the NI titers were comparable for N2 but lower than the rgH6N1 control. As in Fig. 3f, challenge with ZJ19 only boosted antibodies to AI68 and its homologous antigen (Fig. 4c and d). Except for mice in the PBS group, all animals survived the challenge (Supplemental Fig.3d, e, f).

To demonstrate that the antibodies elicited by the AI68 N2 were protective against ZJ19 challenge, we immunized another set of mice and conducted passive transfer of the immune sera into naïve mice prior to challenge (Fig.4a(ii)). Mice that received SG16 and MI15 immune sera lost significantly more weight than mice receiving AI68 N2-primed sera at one to seven days post-inoculation (Fig.4e). After day eight, surviving mice recovered their weight and were mostly indistinguishable from mice immunized with ZJ19. Despite some early weight loss, 70% of mice that received sera from AI68-primed mice survived compared to 0- 25% survival rate in the other groups (Fig.4f). These data indicate that N2 antibodies generated by AI68 priming were protective against lethal A(H9N2) AIV infection compared to contemporary human N2."

4. Lines 50-51: "Seasonal A(H3N2) infection could recall these AIV N2 antibodies, showing distinct human- or avian NA-type reactivity, in 19-26% of individuals."
I did not find these estimates from the main text. 19-26% of whom? All age groups? Please clarify.

Response: We thank the reviewer for pointing this out. The 19-26% was referring to the data in Table 2, whereby 19% of individuals who seroconverted to N2 of A/Hong Kong/4801/2014 (H3N2) also seroconverted to A/guinea fowl/Hong Kong/WF10/1999 (H9N2) (HK99) and 26% to A/Pakistan/486/2015 (H9N2) (PA15). This percentage was referenced in manuscript on Page 13, Line 271 as "Of this group, seroconversion rates were 39.5% to AI68, 18.6% to HK99, 25.6% to PA15, 18.6% to N3, 2.3% to N7, 4.7% to N9 and 11.6% to N5 (Table 2), displaying a similar trend in cross-reactivity as seen with the baseline sera." We rounded up this figure in the original abstract to help with readability. However, we have removed this value from the revised abstract to comply with word limit.

5. Lines 246-248: 'N3 and N9 titers were also highest among 60-69yo, with titers significantly higher compared to younger adults.' Please discuss this in the Discussion section.

Response: We have included a discussion on this point in the discussion section as suggested by the reviewer (see below). Presently, we do not have a good explanation for this observation. Because the N9 and N3 titers correlated with avian NA titers (Figure 5i) within this narrow age range, this suggests that it may be related to avian-type NA reactivity, potentially conferred by exposure to the A(H2N2) pandemic.

Page 17, Line 358-361:
"Because the N9 and N3 titers correlated with avian NA titers (Fig.5i) and was observed only within this narrow age range, it may be related to avian-type NA reactivity, potentially conferred by exposure to the H2N2 pandemic, although this needs to be tested experimentally."

Reviewer #3 (Remarks to the Author):

Imprinting is known to exert profound population-level effects on the outcome of subsequent influenza virus infections (and vaccinations). Yet, the vast majority of data in the field has focused on imprinting by HA (at the exclusion of NA). NA-specific antibodies are known to confer protection from influenza virus infection, and NA is more immunogenic than HA on a molecule-per-molecule basis. The manuscript by Liang et al explores NA-specific imprinting conferred by the 1957 H2N2 pandemic and the 1968 H3N2 pandemic, and the impact of this imprinting on immunity to contemporary avian H9N2 viruses.

There is no doubt that this is a very important and potentially impactful topic. The data shown in Fig.2 convincingly show age-specific trends in NA cross-reactivity consistent with what has been previously described in the context of HA. However, the subsequent experiments performed to evaluate whether these NA-specific antibodies are truly cross-protective, and the extent of their recall, suffer from limitations that confound interpretation and limit the strength of conclusions that can be drawn. Specific remarks for the authors' consideration can be found below:

Major Concerns:

1. It is extremely difficult to follow/interpret the data shown in Fig. 3a-d due to a lack of clarity in both the figure legend and corresponding results section. These should be re-written to facilitate easier interpretation. Perhaps inclusion of experimental schema would be helpful.

Response: We apologize for the poor writing in this section. We have revised this section to improve clarity by separating the experiments described in Figure 3 into two parts. The first part describes the antigenic relationship between early human and avian N2 (page 10, Line 190, Figure 3) and the second part demonstrates the protective capacity of AI68 N2 antibodies against A(H9N2) challenge (page 11, Line 213, Figure 4). The first part uses wild-type or wild-type like viruses to study cross-reactivity. In the second part, we wanted to demonstrate in system without interference of HA immunity that the observed cross-reactivity can be protective. For this second part, we generated isogenic 7+1 viruses and only varying the NA segment. In this experiment, ZJ19 was the only wild-type virus used. Further, we included new experimental schema for each experiment (Figure 3a and Figure 4a(i) and (ii)), clearly indicating what viruses were used in the Figures and Figure legends.

The revisions are as follows:

Page 10-11, Line 190-231:
"Antigenic relationship between early human and avian N2.
To determine if exposure to early human N2s was a determinant of cross-reactivity, we performed a prime-challenge experiment in mice (Fig.3a). Mice was primed with A/Aichi/2/1968(H3N2) (AI68) wild type virus, and reverse-genetics derived viruses bearing the HA and NA of A/Singapore/INFIMH160019/2016 H3N2) (SG16) or A/Michigan/45/2015 (H1N1) (MI15). AI68

chosen to represent the early H3N2 virus while SG16 chosen to represent a contemporary H3N2 virus. MI15 was used as heterologous NA subtype control. Although the priming doses were predetermined to elicit comparable N2 titers between AI68 and SG16 (data not shown), in the eventual experiment, the two priming doses elicited higher homologous HI and NI titers in the AI68-primed mice compared to SG16 (Fig.3b). The control ZJ19 and rgH1N1 viruses had higher HI but lower NI titers compared to AI68.

We assessed the baseline cross-reactivity in the pre-challenge immune sera to the NA of ZJ19. Apart from the positive control, AI68 had the highest titers to ZJ19 and was significantly higher compared to SG16 and MI15 (Fig.3c). Post challenge, AI68- and ZJ19-primed groups showed smaller increases in ZJ19 N2 titers compared to SG16-, MI15- and PBS-primed groups (Fig.3c and d), likely due to the higher levels of baseline antibody. However, only mice in the AI68 or ZJ19 group showed significant induction of priming N2 antibodies and at least four-fold increases in NI-titers after challenge (Fig.3e and f, Supplemental Table 6). All animals were protected, with no significant differences in weight loss amongst groups (Supplemental Fig.3a, b, c), except for those in the PBS group. These data indicate that AI68 and ZJ19 shared cross-reactive epitopes and were antigenically closer compared to SG16.

AI68 immune sera were protective against ZJ19 challenge in mice.
To clearly demonstrate the role of NA and to reduce potential interference from the different HAs, we generated a complementary set of isogenic, reverse-genetics (rg)H6Nx viruses bearing the NA from AI68, SG16, or MI15 in the second prime-challenge experiment (Fig.4a(i)). After two priming doses, the HI titers were comparable amongst the rgH6Nx viruses but were significantly lower than the H9N2 control (Fig.4b) whereas the NI titers were comparable for N2 but lower than the rgH6N1 control. As in Fig. 3f, challenge with ZJ19 only boosted antibodies to AI68 and its homologous antigen (Fig. 4c and d). Except for mice in the PBS group, all animals survived the challenge (Supplemental Fig.3d, e, f).

To demonstrate that the antibodies elicited by the AI68 N2 were protective against ZJ19 challenge, we immunized another set of mice and conducted passive transfer of the immune sera into naïve mice prior to challenge (Fig.4a(ii)). Mice that received SG16 and MI15 immune sera lost significantly more weight than mice receiving AI68 N2-primed sera at one to seven days post-inoculation (Fig.4e). After day eight, surviving mice recovered their weight and were mostly indistinguishable from mice immunized with ZJ19. Despite some early weight loss, 70% of mice that received sera from AI68-primed mice survived compared to 0- 25% survival rate in the other groups (Fig.4f). These data indicate that N2 antibodies generated by AI68 priming were protective against lethal A(H9N2) AIV infection compared to contemporary human N2."

2. In Fig. 3f, the authors rely on the conventional 4-fold change in titers as an indicator of seroconversion to support their argument regarding cross-reactivity of AI68 and ZJ19 N2. However,

the absolute difference relative to SG16, or even MI15 is only slightly greater than 2-fold, which is hardly convincing.

Response: We thank the reviewer for this comment. We would like to clarify that we are not only relying on the conventional 4-fold change in titers as being significant. As indicated in the table below (now included as Supplemental Table 6), all mice in the H3N2-AI68 and rgH6N2-AI68 group showed at least 4-fold increase in antibody titer after challenge with ZJ19, comparable to the positive control group, ZJ19. This is in stark contrast to the responses showed in the other priming groups, which showed less than 2-fold increases in antibody titers. This led to our conclusion of an antigenic relationship between NA of AI68 with ZJ19. We have amended the manuscript to include Supplemental Table 6 for improved clarity, and revisions at page 9, line 180-182, as shown below.

Supplemental Table 6 |Fold change of NI antibody titers against priming viruses after challenge with ZJ19.

Immunization group	H3N2-AI68	rgH3N2-SG16	rgH1N1-MI15	rgH6N2-AI68	rgH6N2-SG16	rgH6N1-MI15	H9N2-ZJ19
Fold change	4	1	0.5	4	0.5	1	8
	8	1	1	8	1	1	4
	4	2	2	4	2	1	4
	4	1	0.25	8	2	0.5	2
	4	2		4	2	1	2

Page 9, Line 180-182: However, only mice in the AI68 or ZJ19 group showed significant induction of priming N2 antibodies and at least four-fold increases in NI-titers after challenge (Fig.3e and f, Supplemental Table 6).

3. Much of the data presented by the authors is potentially confounded by antibodies that may recognize HA. The authors perform HAI assays in several figures, however, antibodies that bind to the HA stalk are typically HAI-negative. Thus, it would be important to measure cross-reactive antibodies by either ELISA (to measure total cross-reactive binding), or a functional assay (for example, one that measures Fc-dependent effector functions) to evaluate their possible contribution. This concern is amplified by the fact that H6 and H9 are both group 1 HAs with highly conserved stalk domains. This confounder could be eliminated completely where the authors to immunize animals with purified, recombinant NA in several experiments.

Response: We thank the reviewer for this insightful comment. We have now included data on HA-stalk antibodies to Group 1 and 2, as well as H9 binding antibodies that were assayed by ELISA on the human and mice serum samples used. In summary, all the human and mice sera samples showed low level of anti-stalk antibodies (Supplemental Fig. 1 and 5). As for the H9-binding antibodies, adults older than 21 yo did show detectable H9-antibodies, but the average titers were not significantly different across the ages of 21-39 yo, 40-64 yo and ≥ 65 yo. This data suggest that H9-

binding antibodies did not play a major role in protection since sera from the 21-39 yo did not protect mice during the challenge experiment (Figure 2g).

During the early phases of study design, we had considered testing our hypothesis by immunizing mice with purified, recombinant NA. However, after reviewing available literature, we found that protein immunization does have its own limitation; as it required a substantial amount of stabilized protein or adjuvants for effective immunogenicity. As we did not have access to such constructs, we chose to use rg-viruses, to which we argue, will also present the NA antigen in a conformation that will mimic those presented during an infection; and more accurately represent what is present in vivo.

Unfortunately, after all the serology and passive transfer experiments, we did not have sufficient sera leftover from the Guangzhou cohort for many assays. Thus, we prioritized our experiments to measure HA-stalk and H9-binding antibodies. We posit that any HA cross-reactive antibodies elicited should also be detected by H9 and HA-stalk ELISAs. Our additional data from both these assays suggest that they do not play a major role in the cross-reactive and protective response observed here. These changes were incorporated into the revised manuscript as a new section:

These changes have been incorporated into the manuscript as an additional result section and as Supplemental Figure 5:

Page 14, Line 287:
"Role of HA-stalk and HA-binding antibodies
HA-stalk antibodies have been reported to interfere with NI assays through steric hindrance[21]. To exclude the potential interference by HA-stalk antibodies in our data, we measured the level of antibodies specific for Group 1 and 2 HA-stalk regions using previously described constructs, pH1HA10-Foldon (designed from A/California/04/2009 (H1N1)) and HK68-H3-SI (designed from A/Hong Kong/1/1968 (H3N2)) [22-24] in the human and mice serum samples used in our study. Minimal antibodies were detected against both stalk proteins across all age groups in both the Guangzhou and CARES cohorts with no significant difference detected across any age groups (Supplemental Fig.5a, b). We also tested the sera from the mouse immunization experiments for HA-stalk, H9 and H6 antibodies. As expected, since H6 belongs to Group 1, mice immunized with the rgH6Nx constructs elicited more Group 1 HA stalk antibodies compared to Group 2, although the titers were still relatively low. No significant difference of anti-HA stalk antibodies and anti-H9 binding were detected across the different groups of immunized mice (Supplemental Fig.5 c, d). No cross-reactivity were observed between H6 and H9 (Supplemental Fig.5d). We conclude that HA-stalk and H9-binding antibodies are not major determinants of the cross-reactivity observed."

Additionally, to exclude the potential interference by H9-binding antibodies on the cross-reactive NI activity, we re-analyzed our data using only individuals who were H9-seronegative by HAI and ELISA. Re-analyses of the Guangzhou Cohort is included as Supplemental Figure 2 and for the CARES cohort as Supplemental Figure 4 and Supplemental Table 8. In both re-analyses, our original pattern of cross-reactivity and its correlations still stands. This point is incorporated into our revised manuscript as follows:

For data describing Guangzhou Cohort, Page 9, Line 178: "To further exclude the role of HA-binding antibodies in our observation, whether from past H9N2 exposure or any cross-reactive antibodies that may target H9, we measured their H9-binding antibodies against recombinant H9 protein A/Hong Kong/1073/99 (H9N2) by ELISA. Some individuals above the ages of 21 showed H9-binding positivity (Supplemental Fig.1a). However, except for those aged ≥ 65yo compared to the 11-20yo and the ≤ 5yo, there were no statistically significant difference amongst other age groups. Because pooled sera from the 21-39 yo did not protect the mice from the ZJ19 challenge (Fig 2f, g), this suggests that H9-binding antibodies were not a major determinant in the protective effect observed. Further, even after excluding those participants with anti-H9 binding positivity, all our observed pattern of cross-reactivity in the Guangzhou cohort still holds (Supplemental Fig.2), suggesting that this avian N2 cross-reactivity is not due to previous exposures to H9N2."

For data describing the CARES cohort, Page 14, Line 282: "Presence of cross-reactive H9 antibodies at baseline, as determined by either ELISA and HAI, did not seem to affect these NA responses as these differences and correlations still holds even when we restricted the analyses to the 29 H9-seronegative (by both assays) individuals (Supplemental Fig.4, Supplemental Table 8)."

4. In Fig.4, the authors claim that mice that received sera from rgH6N2-AI68 lost less weight than those that received sera from rgH6N2-SG16 however, this is not clear from the data shown in Fig.4a where there seems to be near identical weight loss and overlapping error bars. Indeed, the figure legend seems to indicate that statistical differences shown are relative to the PBS control group. In Fig. 4b the is evidently a difference between the two groups in terms of survival, but these could very well be stochastic given the similarity in the weight loss curves (since all animals appear to lose 20+% of initial body weight. Were 20% weight loss applied as the threshold for endpoint (which is the standard applied by many animal ethics committees), it does not seem that there would be any difference at all in survival.

Response: We thank the reviewer for raising this very salient technical point. We applied 30% weight loss as the humane endpoint (Methods section, page 25, Line 545). Only three out of 10 mice in the rgH6N2-AI68 group reached this point vs. 6 out of 8 mice in the rgH6N2-SG16 group. This was supported statistically when we reanalyzed the survival rate of Fig. 4f using SG16 as comparator (see figure below). It is unlikely that there is any stochastic effect in this data.

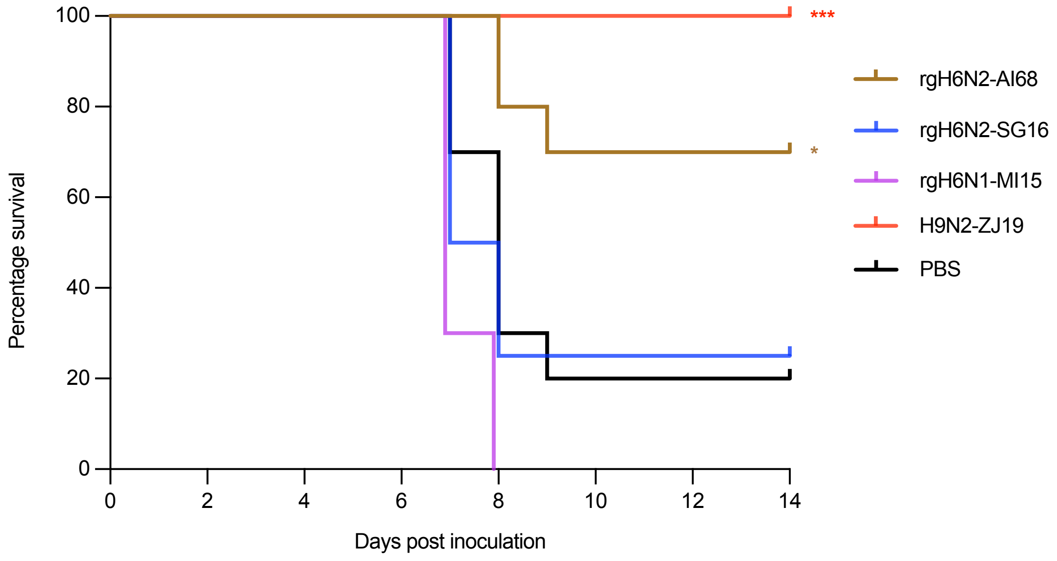

100
80
60
40
20
0
Percentage survival
0
2
4
6
8
10
12
14
Days post inoculation
***
*
rgH6N2-AI68
rgH6N2-SG16
rgH6N1-MI15
H9N2-ZJ19
PBS

References

1. Mallajosyula, V.V., *et al.* Influenza hemagglutinin stem-fragment immunogen elicits broadly neutralizing antibodies and confers heterologous protection. *Proc Natl Acad Sci U S A* **111**, E2514-2523 (2014).
2. Sutton, T.C., *et al.* Protective efficacy of influenza group 2 hemagglutinin stem-fragment immunogen vaccines. *NPJ Vaccines* **2**, 35 (2017).
3. Valkenburg, S.A., *et al.* Stalking influenza by vaccination with pre-fusion headless HA mini-stem. *Sci Rep* **6**, 22666 (2016).
4. Arevalo, P., McLean, H.Q., Belongia, E.A. & Cobey, S. Earliest infections predict the age distribution of seasonal influenza A cases. *Elife* **9**(2020).
5. Wong, S.S., *et al.* Hemagglutinin and Neuraminidase Antibodies Are Induced in an Age- and Subtype-Dependent Manner after Influenza Virus Infection. *J Virol* **94**(2020).
6. Wong, S.S., *et al.* Severe Influenza Is Characterized by Prolonged Immune Activation: Results From the SHIVERS Cohort Study. *J Infect Dis* **217**, 245-256 (2018).

Reviewers' Comments:

Reviewer #1:
Remarks to the Author:
The authors have addressed the reviewers' comments well.

Reviewer #3:
Remarks to the Author:
Overall, the authors have done a considerable amount of work to address the points raised by reviewers. As noted during the original submission, this is an interesting and important topic.

The authors spent considerable effort measuring HA stalk antibody titers, an issue that was raised by all reviewers. While the approach taken to rule out the contribution of these antibodies to NI cross-reactivity was less direct than may have been ideal, addition of the new data provides important added context for readers.

This reviewer remains somewhat skeptical of the robustness of the survival data in Fig.4f using a 30% vs 20% weight loss threshold for endpoint. Nevertheless, the accompanying weight loss curves provide sufficient data transparency that readers can make judgements for themselves. Ultimately, these mouse models are imperfect and the reviewer appreciates the experimental approach.
